# Anti-inflammatory effects of moxifloxacin and levofloxacin on cadmium-activated human astrocytes: Inhibition of proinflammatory cytokine release, TLR4/STAT3, and ERK/NF-κB signaling pathway

**DOI:** 10.1371/journal.pone.0317281

**Published:** 2025-01-14

**Authors:** Suttinee Phuagkhaopong, Jidapha Sukwattanasombat, Kran Suknuntha, Christopher Power, Piyanuch Wonganan, Pornpun Vivithanaporn

**Affiliations:** 1 Department of Pharmacology, Faculty of Medicine, Chulalongkorn University and King Chulalongkorn Memorial Hospital, Bangkok, Thailand; 2 Interdisciplinary Program in Pharmacology, Graduate School, Chulalongkorn University, Bangkok, Thailand; 3 Chakri Naruebodindra Medical Institute, Faculty of Medicine Ramathibodi Hospital, Mahidol University, Samut Prakan, Thailand; 4 Department of Medicine, University of Alberta, Edmonton, Alberta, Canada; Lady Hardinge Medical College, INDIA

## Abstract

Cadmium is a non-essential element and neurotoxin that causes neuroinflammation, which leads to neurodegenerative diseases and brain cancer. To date, there are no specific or effective therapeutic agents to control inflammation and alleviate cadmium-induced progressive destruction of brain cells. Fluoroquinolones (FQs), widely used antimicrobials with effective blood-brain barrier penetration, show promise in being repurposed as anti-inflammatory drugs. Therefore, we aimed to test the efficacy of repurposed FQs for the treatment of cadmium-induced inflammation using cultures of U-87 MG human astrocytes and primary human astrocytes. Both FQs abrogated cadmium-induced interleukin (IL)-6 and IL-8 release from human astrocytes in a concentration and time-dependent manner, although levofloxacin had a stronger inhibitory effect than moxifloxacin. The downregulation of inflammatory cytokine release occurred with a concomitant reduction in cadmium-induced elevations in p65 nuclear factor-κB (NF-κB) and extracellular signal-regulated kinases (ERKs) 1/2 phosphorylation. Additionally, levofloxacin treatment significantly alleviated cadmium-induced activation of phosphorylated NF-κB translocation and toll-like receptor (TLR)-4/signal transducer and activator of transcription (STAT) 3 signaling. Transcriptome analysis revealed that modulation of inflammation-related pathways was the most enriched after FQ treatment. Our data suggest that FQs, particularly levofloxacin, attenuate the inflammatory process mediated by cadmium in human astrocytes. These effects may be mediated, at least in part, by inhibition of immune pathways regulated by TLR4, STAT3, ERK MAPK, and NF-κB.

## Introduction

Cadmium is a non-essential element classified as a human carcinogen by the International Agency for Cancer Research and the US Environmental Protection Agency; cadmium intoxication is becoming a global health problem [1]. Prolonged exposure to cadmium is strongly correlated with a wide range of neurological issues, such as malignancy, as well as long-term motor and cognitive impairment in children [2, 3] and mining workers [4, 5]. Astrocytes, glial cells of the central nervous system (CNS), are the brain’s most abundant and proliferative cell type. They play an essential role in the defense of neurons against neurotoxic insults; therefore, they are a primary target in mitigating heavy metal neurotoxicity. Although there are no cadmium-specific transport channels, cadmium enters cells via transport by other essential element channels (Ca^2+^, Fe^2+^, and Zn^2+^) [6]. Cadmium also penetrates the brain via the blood–brain barrier (BBB) and olfactory pathways; once cadmium has entered the brain, it causes irreversible damage due to its very long biological half-life (10 to 30 years) [7]. Excessive accumulation of cadmium in astrocytes triggers long-lasting inflammatory responses. We previously showed that although interleukin (IL)-6 and IL-8 are barely detectable in healthy astrocytes, they are rapidly induced 10- to 100-fold in response to cadmium [8], with even 1 μM cadmium, about 50 times below the safe level of cadmium intake recommended by the United States’ Food and Drug Administration (45 μM), eliciting an inflammatory response.

Dysregulation of immune and inflammatory responses in the CNS microenvironment is another way by which cadmium exerts neurotoxic effects; this dysregulation may contribute to the development or progression of brain cancer and glioblastoma (GBM), the most common and aggressive malignant brain tumor in adults [9]. IL-6 and IL-8 likely play pivotal roles in microenvironment-associated chemotherapeutic resistance in GBM [10]; therefore, using anti-inflammatory drugs in addition to cytotoxic drugs may result in more successful GBM treatments. Unfortunately, there are no proven effective treatments for chronic cadmium intoxication. One of the major difficulties in treating cadmium poisoning in the brain is the presence of the BBB, because most chelator agents, such as ethylenediaminetetraacetic acid (EDTA), cannot cross the BBB under physiological conditions [11].

Fluoroquinolones (FQs) are broad-spectrum quinolone antibiotics commonly used in clinical practice for urinary tract infections; FQs have a well-established pharmacokinetic and safety profile with a wide therapeutic index and minimal serious side effects [12]. FQs also exert pleiotropic immunomodulatory effects. A previous experiment indicated that levofloxacin could be repurposed as a therapy to treat inflammation resulting from chronic bacterial infections such as pneumonia and cystic fibrosis [13]. Several studies in immune cells i.e., human peripheral blood monocytes and THP-1 macrophages, show that FQs possess immunomodulatory properties and affect the levels of multiple molecules, including inducible nitric oxide synthase, cyclooxygenase-2, and other pro-inflammatory mediators [14–17]. However, to date, the anti-neuroinflammatory activities of FQs have not been explored. Moxifloxacin, levofloxacin, and ciprofloxacin are considered a preferred choice for the treatment of bacterial meningitis and tuberculous meningitis due to their excellent lipid solubility, compared to norfloxacin, ofloxacin, and gemifloxacin [18]. The therapeutic concentrations in cerebrospinal fluid (CSF) of these three drugs can be achieved after oral or intravenous administration. The median area under the curve (AUC) CSF/AUC serum ratios of moxifloxacin, levofloxacin, and ciprofloxacin were 0.53, 0.44, and 0.24, respectively [19, 20]. However, some studies have shown that ciprofloxacin, a second-generation FQ, has been associated with a higher risk of tendonitis compared with newer-generation FQs such as moxifloxacin and levofloxacin [21]. Moreover, ciprofloxacin is classified as a weak base and needs an acidic environment to dissolve [22]. The study of ciprofloxacin in cell culture models is challenging because acidic pH may affect the stability and efficacy of the drug; we, therefore, did not include ciprofloxacin in this study.

The neuroprotective effects of FQs are intriguing in preclinical studies; however, clinical evidence is still being developed. Moxifloxacin, levofloxacin, and ciprofloxacin are the most studied FQs for potential neuroprotective effects. Two published case reports elicited benefit outcomes of levofloxacin administration in patients diagnosed with Parkinson’s disease [23] and progressive supranuclear palsy (PSP) [24]. Another phase 2a proof-of-concept study in patients with amyotrophic lateral sclerosis (ALS) who received three daily doses of combined ciprofloxacin and celecoxib (PrimeC) for one year confirmed the safety and efficacy of the drug for ALS treatment [25]. Ex vivo CSF and serum samples from meningitis patients given a single oral dose of 400 mg moxifloxacin influence the production of pro- and anti-inflammatory cytokines in lipopolysaccharide-activated human monocytes [14]. However, there is a need for more robust clinical trials to understand their mechanisms of action better.

In the present study, we explored the mechanisms underlying the anti-neuroinflammatory effect of FQs during cadmium poisoning. We demonstrate that moxifloxacin and levofloxacin inhibit cadmium-induced secretion of IL-6 and IL-8 in human astrocytes and that FQs selectively reduce cadmium-induced nuclear factor-κB (NF-κB), extracellular signal-regulated kinases (ERKs), toll-like receptor (TLR)-4, and signal transducer and activator of transcription (STAT) 3 expression or activation. Our results suggest that inhibition of these pathways may mediate the anti-neuroinflammatory effect of FQs.

## Materials and methods

### Human astrocyte cultures

Primary human astrocytes (PHAs) were provided by Prof. Christopher Power (Department of Medicine, University of Alberta, Canada). PHAs were obtained from 15–19 week aborted fetuses over a five-year period May 2018 to May 2023 with written consent under protocol 27660 approved by the University of Alberta Human Research Ethics Board (Biomedical) and were prepared as reported previously [26]. PHAs were grown in Minimum Essential Medium (MEM) containing 10% fetal bovine serum, 1% L-glutamine, 1% sodium pyruvate, and 1% penicillin–streptomycin (Gibco, NY, USA). The human astrocytoma U-87 MG cell line (U-87 MG) was obtained from American Type Culture Collection (VA, USA) and cultured in MEM supplemented with 10% fetal bovine serum, 1% sodium pyruvate, and 1% penicillin–streptomycin. Cells were grown at 37°C in 5% CO_2_ fully humidified air and subcultured twice weekly. Cells were used from the fifth to the tenth passage. All biological reagents used in cell culture are guaranteed mycoplasma-free.

### Preparation of cadmium chloride, moxifloxacin, and levofloxacin

Cadmium chloride (CdCl_2_), moxifloxacin, and levofloxacin were purchased from Sigma Aldrich (MO, USA). Cadmium chloride was dissolved in sterile water at a concentration of 1000 mM and stored at −20°C. Moxifloxacin and levofloxacin were freshly dissolved in sterile water at a concentration of 10 mM and 20 mM, respectively. In the experiments, stock solutions were diluted in serum-free MEM (MEM plus 1% sodium pyruvate) to reach the indicated concentrations.

### MTT cell viability assay

PHAs and human astrocytoma U-87 MG cells were plated at 1.5 × 10^4^ cells/well in a 96-well plates in complete MEM for 24 h followed by exposure to moxifloxacin, levofloxacin, cadmium, or combinations of moxifloxacin or levofloxacin with cadmium, in serum-free MEM for 24 h before assessment of cell viability by MTT assays. MTT solution was added to each well to a final concentration of 0.5 mg/mL. Formazan crystals were dissolved in dimethylsulfoxide and measured spectrophotometrically at 570 and 630 nm using a microplate reader (Thermo Fisher Scientific, Vantaa, Finland). Viability in treated cells was calculated as a percentage of that in mock-treated with culture medium.

### Measurement of intracellular cadmium

Human astrocytoma U-87 MG cells were plated at 1.5 × 10^6^ cells in 100 mm culture dishes in complete MEM for 24 h followed by exposure to cadmium, or combinations of moxifloxacin or levofloxacin with cadmium, in serum-free MEM for 24 h. Cells were then washed twice with phosphate-buffered saline (PBS) containing 10 mM disodium EDTA and twice with PBS without EDTA, trypsinized with 0.1% trypsin, and centrifuged at 3,000 × g for 10 min. Cell pellets were digested with concentrated nitric acid (65% w/w, 1 mL) at 90°C three times before digesting with concentrated nitric acid (65% w/w, 2 mL) overnight at room temperature. Afterward, the digested samples were diluted to a final volume of 20 mL and cadmium contents were measured using a flame furnace atomic absorption spectrophotometer (PinAAcle 900T, Perkin Elmer, MA, USA). The amount of intracellular cadmium content was expressed as μg of cadmium/g of wet weight protein.

### Enzyme-linked immunosorbent assay (ELISA)

Human astrocytoma U-87 MG cells were plated at 1.2 × 10^5^ cells/well in 12-well plates in complete MEM for 24 h followed by exposure to moxifloxacin, levofloxacin, or cadmium, or combinations of moxifloxacin or levofloxacin with cadmium, in serum-free MEM for 6 or 24 h. Spent media from U-87 MG cells was then collected and the amount of human IL-6 and IL-8 was determined using hIL-6 and hIL-8 ELISA kits according to the manufacturer’s instructions (eBioscience, CA, USA). Briefly, samples containing IL-6 and IL-8 were added to ELISA plates precoated with purified human IL-6 and IL-8 antibodies. IL-6 and IL-8 proteins linked with IL-6 and IL-8 antibodies were labeled with a biotin-conjugated antibody, avidin-HRP complex. This complex was then detected with the 3,3′, 5,5′-tetramethylbenzidine peroxidase solution and measured spectrophotometrically at 450 and 570 nm using a microplate reader (Thermo Fisher Scientific, Vantaa, Finland). The concentrations of the samples were calculated from the standard curve and expressed as pg/mL.

### Western blotting

PHAs and human astrocytoma U-87 MG cells were plated at 5 × 10^5^ cells in 60 mm culture dishes in complete MEM for 24 h followed by exposure to cadmium, or combinations of moxifloxacin or levofloxacin with cadmium, in serum-free MEM for 1 h for detection of NF-κB and ERK or 3h for detection of TLR4 and STAT3. Cells were then washed with cold PBS and exposed to lysis buffer (50 mM Tris-base, 150 mM NaCl, 0.5% Nonidet P-40 in addition to protease inhibitor cocktail). Cell debris was removed by centrifugation and the supernatants were collected as whole-cell lysates. Total protein was quantified using Bio-Rad DC Protein assay reagents (Bio-Rad, Hercules, CA, USA). Proteins (10 μg) were separated using 12% SDS-PAGE and electroblotted in 20% methanol, 25 mM Tris, and 192 mM glycine onto a nitrocellulose membrane. The membrane was blocked with 5% nonfat dry milk in 25 mM Tris-HCl, 150 mM NaCl, and 0.1% Tween 20, followed by incubation overnight at 4°C with anti-ERK1/2 (Cat# 4695 diluted 1:1000—Cell Signaling, MA, USA), anti-p-ERK1/2 (Cat# 3179 diluted 1:1000—Cell Signaling), anti-p65 NF-κB (Cat# AB16502 diluted 1:1000—Abcam), anti-p-p65 NF-κB (Cat# AB86299 diluted 1:1000—Abcam, Cambridge, UK), anti-STAT3 (Cat# 12640s diluted 1:1000—Cell Signaling, MA, USA), anti-p-STAT3 (Cat# 9145s diluted 1:2000—Cell Signaling, MA, USA), anti-TLR4 (Cat# 48–2300 diluted 1:200—Invitrogen, MA, USA), or β-actin (Cat# 4070 diluted 1:3000—Cell Signaling, MA, USA), or GAPDH (Cat# 5174 diluted 1:3000—Cell Signaling, MA, USA). Afterward, membranes were incubated at room temperature for 2 h with monoclonal anti-rabbit IgG-HRP (Cat# 111-035-003 diluted 1:5000—Jackson ImmonoResearch, PA, USA) and developed using an enhanced chemiluminescence system kit (Thermo Fisher Scientific, MA, USA). Membranes were imaged with Image Studio software version 5.2 (LI-COR, Lincoln, NE, USA). Protein levels were normalized to β-actin or GAPDH.

### Immunofluorescence analysis of NF-κB (p65) localization

Human astrocytoma U-87 MG cells were plated at 1.5 × 10^4^ cells in an 8-well cell culture chamber slide in complete MEM for 24 h followed by exposure to cadmium, or combinations of moxifloxacin or levofloxacin with cadmium, in serum-free MEM for 1 h for detection of NF-κB nuclear translocation. Cells were then washed with cold PBS, fixed with cold 4% paraformaldehyde, permeabilized with 0.1% Triton X-100 (Sigma, MO, USA), and blocked with 1% Bovine serum albumin (Himedia, Maharashtra, India). Subsequently, cell samples were incubated overnight at 4°C with a primary antibody specific to p-p65 NF-κB (Cat# AB86299 diluted 1:500—Abcam, Cambridge, UK), followed by incubation at room temperature for 2 h with monoclonal Alexa fluor 488 goat anti-rabbit IgG-HRP (Cat# A11008 diluted 1:500—Thermo Invitrogen, CA, USA). Fluoroshield mounting medium with DAPI (Cat#104139—Abcam, Cambridge, UK) was counterstained to locate the nucleus. Images were observed by immunofluorescent microscopy (EVOS FL Auto 2 Cell Imaging System—Thermo Invitrogen, CA, USA). Data shown representative of three independent experiments. Original magnification, ×40. The fluorescence intensity of cytoplasmic and nuclear p65 subunit was calculated using ImageJ software and results are presented as a percentage of nucleic over cytoplasmic positive cells to p65 NF-κB.

### RNA preparation and RNA-seq analysis

Human astrocytoma U-87 MG cells were plated at 5 × 10^5^ cells in 60 mm culture dishes in complete MEM for 24 h followed by exposure to cadmium, or combinations of moxifloxacin or levofloxacin with cadmium, in serum-free MEM for 3 h. Cells were then washed with PBS and total RNA was isolated from cells using Monarch Total RNA Miniprep Kit with on-column DNase treatment according to the manufacturer’s instructions (New England Biolabs, MA, USA). An equal amount of RNA was pooled from four independent experiments. The quality and quantity of RNA were analyzed using capillary electrophoresis on the Bioanalyzer 2100 (Agilent Technologies, California, United States). The extracted RNA was stored at −80°C until sequencing. RNA-seq libraries were constructed using the TruSeq mRNA LT Sample Prep Kit using Illumina HiSeq 2500 (Illumina, CA, USA), according to the manufacturer’s instructions, by Macrogen (Seoul, South Korea). RNA-seq analysis was performed by BMKGENE Multi-omics Sequencing Services (Beijing, China). Briefly, high-quality reads were aligned with the human genome hg38 (Homo_sapiens.GRCh38_release95.genome.fa) using HISAT2. Transcripts from the preprocessed RNA-seq alignment assembly were identified, and their expression levels were measured using Stringtie with Gencode V32 annotation. The sequencing data of the following groups were combined for analysis using Stringtie Merge: (1) mock-treated vs. cadmium-treated; (2) cadmium-treated and moxifloxacin vs. cadmium-treated; (3) cadmium-treated and levofloxacin vs. cadmium-treated; and (4) combinations of moxifloxacin with cadmium-treated group vs. combinations of levofloxacin with cadmium-treated group. DESeq2 was then used to compare the combined transcripts to determine the differential expression levels of given transcripts.

### Mapping identification of differentially expressed genes and functional enrichment analysis

AnnotateDESeq2 was used to map transcripts with annotated gene IDs to generate a list of differentially expressed genes (DEGs). Down-regulated genes were defined as those with a log2FC < −1 and P value < 0.05 and up-regulated genes were defined as those with a log2FC > 1 and P value < 0.05. To provide insights into the biological function of enriched DEGs, DEGs in the following groups were subjected to Gene Ontology (GO) term functional enrichment analysis and Kyoto Encyclopedia of Genes and Genomes (KEGG) pathway enrichment analysis using the g:Profiler tool: (1) mock-treated vs. cadmium-treated; (2) cadmium-treated vs. moxifloxacin and cadmium-treated; (3) cadmium-treated groups vs. levofloxacin and cadmium-treated; and (4) combinations of moxifloxacin with cadmium-treated group vs. combinations of levofloxacin with cadmium-treated group. Pathway mapping was conducted using the Homo sapiens Orthology database as a reference.

### Statistical analyses

Statistical analyses were performed using GraphPad Prism software (GraphPad Software, version 10, CA, USA). All results were expressed as means ± standard error of mean (SEM) from at least three independent experiments performed in triplicate. Multiple comparisons were performed by a one-way analysis of variance (ANOVA) followed by Tukey’s post-hoc tests. Student’s t-tests were used to detect significant differences between two groups. A P value < 0.05 was considered statistically significant.

## Results

### Cellular toxicity of FQs on U-87 MG human astrocytes and PHAs

**Fig 1:**
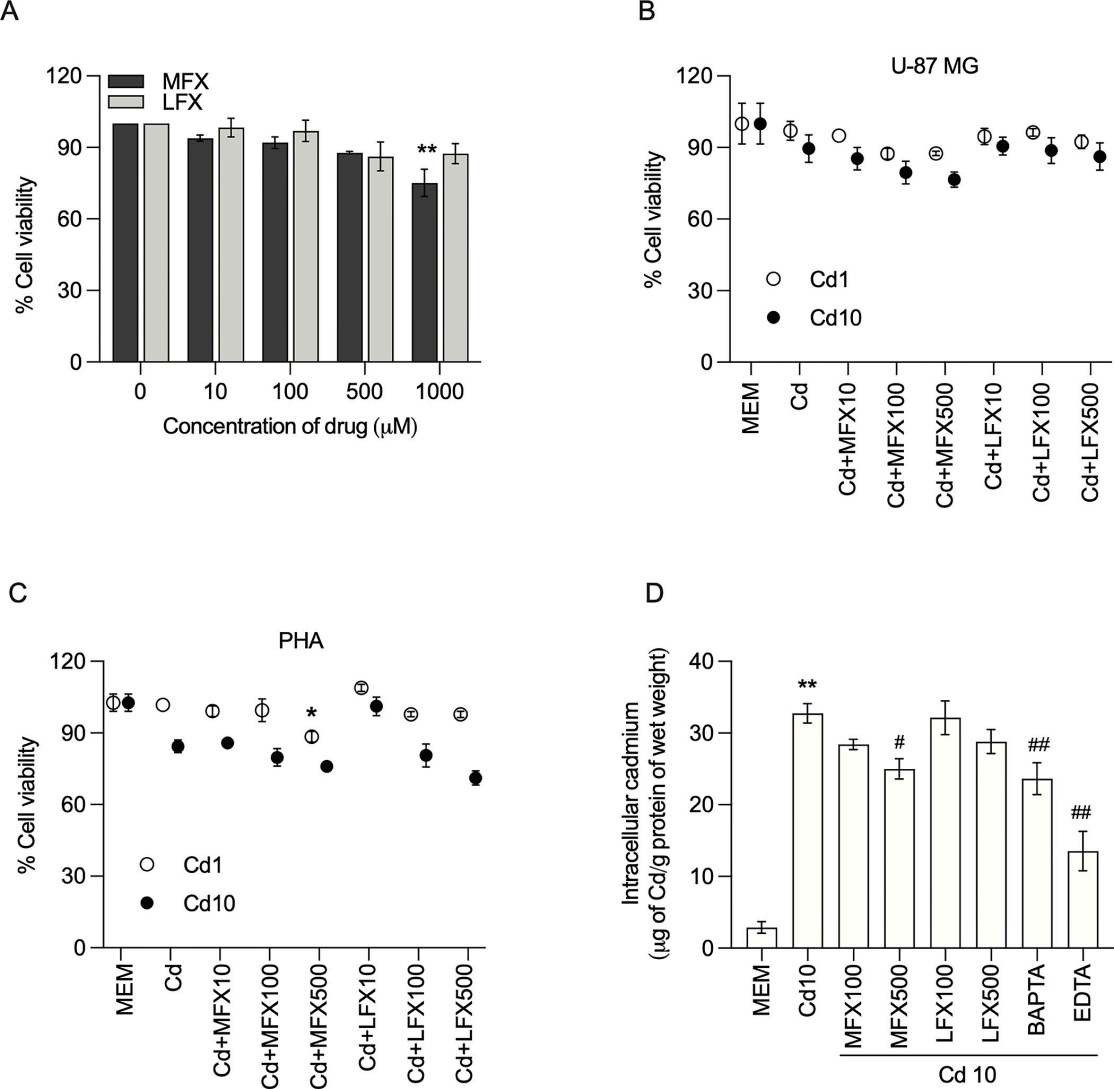
Effect of moxifloxacin and levofloxacin on cell viability of U-87 MG human astrocytes and primary human astrocytes. **A** U-87 MG cells were treated with various concentrations of either moxifloxacin or levofloxacin (0, 10, 100, 500, and 1000 μM) for 24 h and cell viability was determined by MTT cell viability assay. **B, C** U-87 MG cells and primary human astrocytes were cotreated with cadmium (1–10 μM) and either moxifloxacin or levofloxacin (10–500 μM) for 24 h and cell viability was determined by MTT cell viability assay. The cell viability results are expressed as the percentages of mock-treated cells. **D** U-87 MG cells were cotreated with cadmium (10 μM) and either moxifloxacin or levofloxacin (100 and 500 μM) for 24 h and the intracellular cadmium level was determined by FAAS technique. 10 μM of BAPTA and 20 of μM EDTA were used as positive controls. Data represents mean±SEM of at least three independent experiments. *P<0.05 and **P<0.01 compared between cadmium-treated cells and mock-treated cells; ^#^P<0.05 and ^##^P<0.01 compared between cells exposed to cadmium and moxifloxacin or cadmium and levofloxacin compared with cadmium alone. Cd, Cadmium; MFX, Moxifloxacin; LFX, Levofloxacin.

To investigate the anti-inflammatory effects of FQs independent of cell growth inhibition, their potential cytotoxicity on U-87 MG human astrocytes and PHAs was assessed. Cells were incubated with either moxifloxacin or levofloxacin at various concentrations (10, 100, 500, 1000 μM) for 24 h and cell viability was measured using an MTT assay. As shown in **Fig 1A**, there were no significant toxic effects of FQs on cells at concentrations between 10 and 500 μM. When cells were exposed to both cadmium and FQs, cytotoxic effects of FQs were not seen **(Fig 1B)**. Consistent with the results in U-87 MG human astrocytes, FQ administration at all concentrations did not induce significant cell death in PHAs **(Fig 1C)**. In general, intracellular cadmium accumulation in human U-87 MG astrocytes decreased with increasing moxifloxacin and levofloxacin concentrations. At 500 μM, moxifloxacin had a strong chelating effect in the presence of cadmium **(Fig 1D)**. To employ therapeutically relevant concentrations and avoid cytotoxic effects, FQ concentrations of 10 and 100 μM were selected for the following experiments.

### FQs reduce the production of proinflammatory cytokines in cadmium-activated U-87 MG human astrocytes

**Fig 2:**
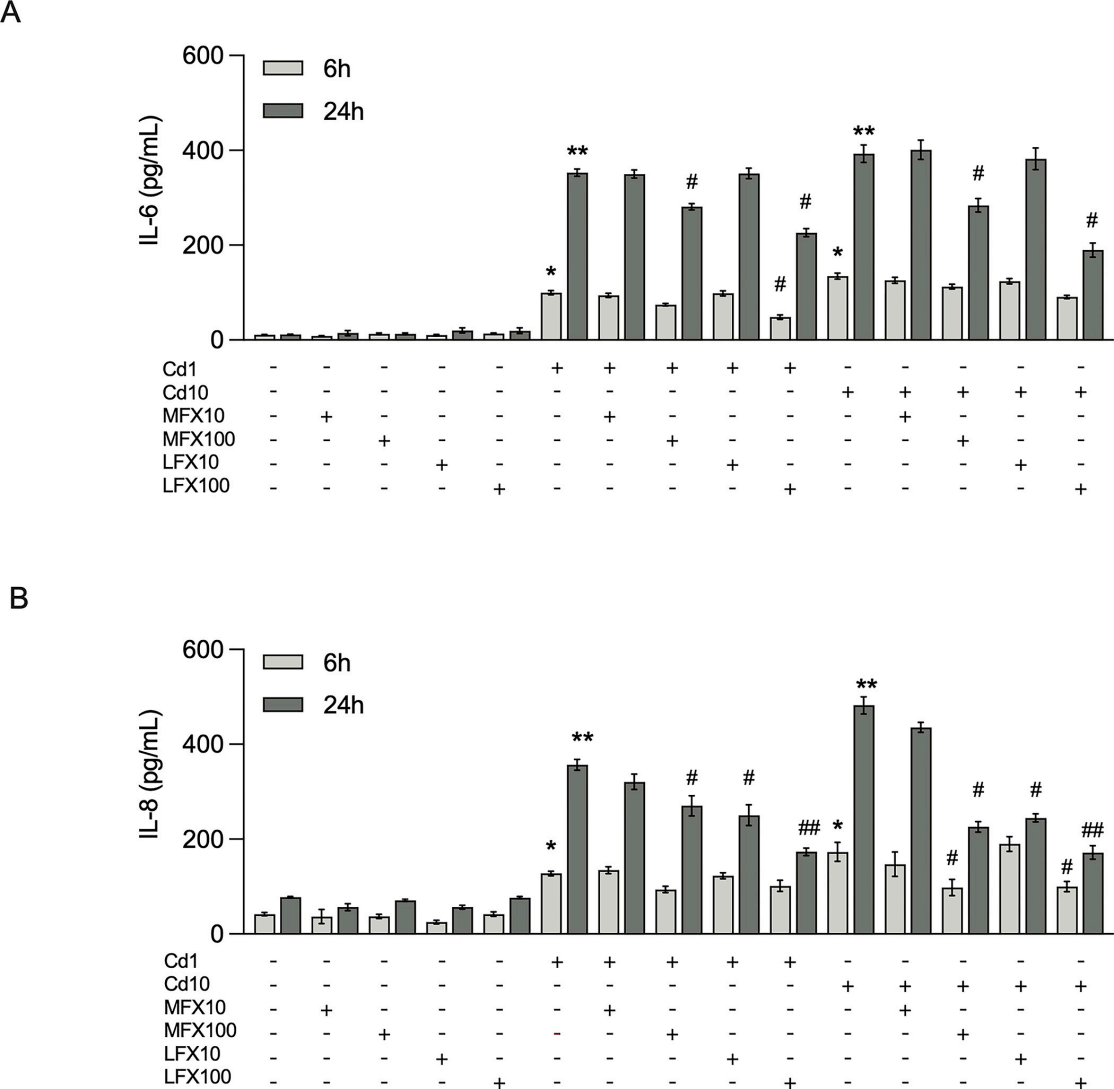
Effect of moxifloxacin and levofloxacin on cadmium-induced upregulation of IL-6 and IL-8 in U-87 MG human astrocytes. **A, B** U87-MG cells were exposed to cadmium (1 and 10 μM) in the absence or the presence of 10 and 100 μM moxifloxacin or levofloxacin for 6 and 24 h and the protein levels of IL-6 and IL-8 in the culture medium were determined by ELISA. Data represents mean±SEM of at least three independent experiments. *P<0.05 and **P<0.01 compared between cadmium-treated cells and mock-treated cells; ^#^P<0.05 and ^##^P<0.01 compared between cells exposed to cadmium and moxifloxacin or cadmium and levofloxacin compared with cadmium alone. Cd, Cadmium; MFX, Moxifloxacin; LFX, Levofloxacin.

To confirm the anti-inflammatory role of FQs in cadmium activation, protein levels of IL-6 and IL-8 in the cell culture medium of U-87 MG cells treated with 1 and 10 μM of cadmium, either in the presence or absence of 10 and 100 μM FQs, were determined by ELISA. As shown in **Fig 2A, 2B**, cadmium dose-dependently induced IL-6 and IL-8 release from U-87 MG cells, while FQs alone did not have any effect on cytokine production. The cadmium-induced increase in IL-6 and IL-8 was ameliorated by FQ treatment in a concentration and time-dependent manner; however, moxifloxacin administered at 10 μM had no significant effects on either IL-6 or IL-8 secretion (both P > 0.05). Overall, moxifloxacin had a smaller inhibitory effect on both IL-6 and IL-8 release than levofloxacin.

### FQs suppress the phosphorylation of ERK and NF-κB in cadmium-activated U-87 MG human astrocytes and PHAs

**Fig 3:**
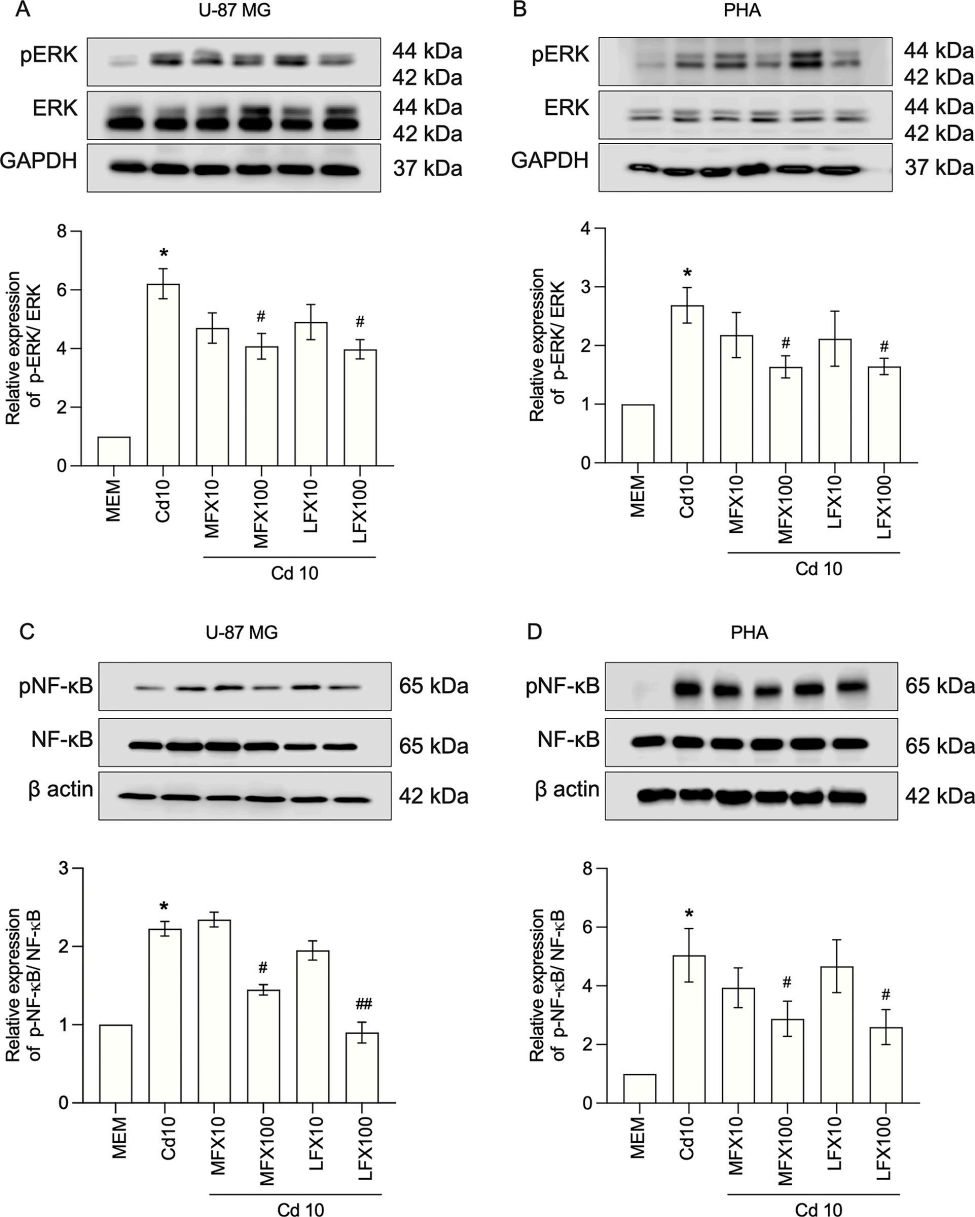
Effect of moxifloxacin and levofloxacin on cadmium-induced ERK and p65 NF-κB phosphorylation in U-87 MG human astrocytes and primary human astrocytes. **A, B** Representative blots of phosphorylated and total forms of ERK1/2 in U87-MG cells and primary human astrocytes exposed to cadmium (10 μM) in the absence or the presence of 10 and 100 μM moxifloxacin or levofloxacin for 1 h. **C, D** Representative blots of phosphorylated and total forms of p65 NF-κB in U87-MG cells and primary human astrocytes exposed to cadmium (10 μM) in the absence or the presence of 10 and 100 μM moxifloxacin or levofloxacin for 1 h. Data represents mean±SEM of at least three independent experiments. *P<0.05 compared between cadmium-treated cells and mock-treated cells; ^#^P<0.05 and ^##^P<0.01 compared between cells exposed to cadmium and moxifloxacin or cadmium and levofloxacin compared with cadmium alone. Cd, Cadmium; MFX, Moxifloxacin; LFX, Levofloxacin. The original blots are presented in S1 Raw images.

**Fig 4:**
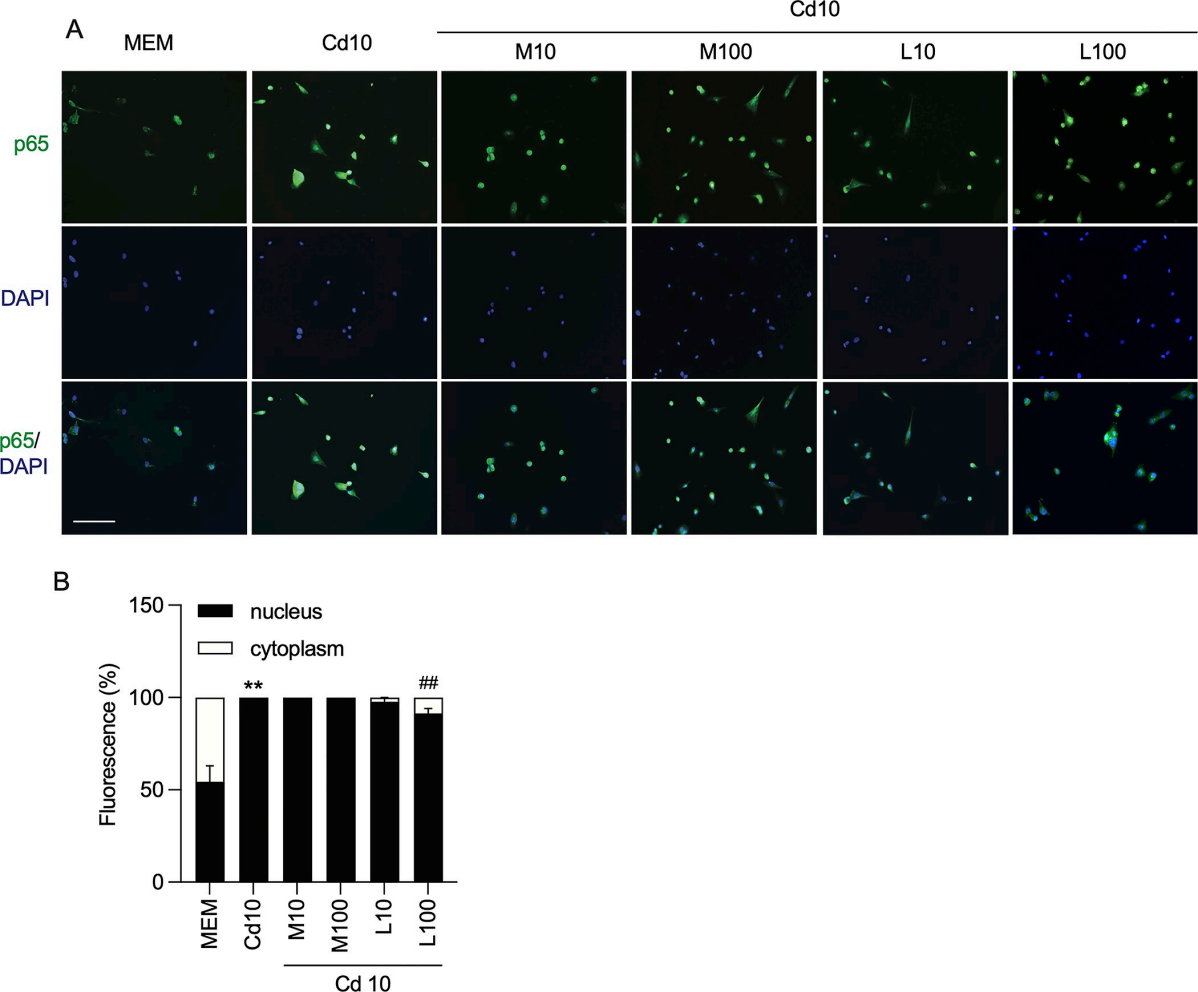
Effect of moxifloxacin and levofloxacin on cadmium-induced p65 NF-κB nuclear translocation in U-87 MG human astrocytes. **A, B** Representative images of immunofluorescence staining of p65 NF-κB and percentage of nucleic over cytoplasmic positive cells to p65 NF-κB in U87-MG cells exposed to cadmium (10 μM) in the absence or the presence of 10 and 100 μM moxifloxacin or levofloxacin for 30 min. The nuclei of astrocytes were labeled by DAPI (blue) and the NF-κB protein was labeled by anti-p65 antibody (green). Cells were photographed under confocal microscopy (scale bar 100 μm, magnification × 40). Data represents mean±SEM of at least three independent experiments. *P<0.05 compared between cadmium-treated cells and mock-treated cells; ^#^P<0.05 and ^##^P<0.01 compared between cells exposed to cadmium and moxifloxacin or cadmium and levofloxacin compared with cadmium alone. Cd, Cadmium; MFX, Moxifloxacin; LFX, Levofloxacin.

As mitogen-activated protein kinase (MAPK) and NF-κB are major regulators of the inflammatory response induced by cadmium, we hypothesized that the anti-inflammatory activity of FQs might result from blocking activation of these pathways. To ensure the robustness of our findings and confirm the observed effects are not confined to a single cell line, we designed the study to incorporate two distinct cells. Western blotting analysis of U-87 MG human astrocytes and PHAs revealed a similar therapeutic response to the drugs. While cadmium-treated cells had significantly higher levels of ERK1/2 and p65 NF-κB phosphorylation than mock-treated cells (P < 0.05), this increase was alleviated by treatment with FQs at 100 μM, but not 10 μM **(Fig 3)**. In particular, the phosphorylation levels of p65 NF-κB were significantly lower in cells treated with cadmium and levofloxacin than in those treated with cadmium and moxifloxacin (P < 0.05) **(Fig 3C, 3D)**. Furthermore, the immunocytochemical analysis confirmed the Western blotting findings that cadmium induced translocation of p65 from the cytoplasm to the nucleus, and levofloxacin appeared to have a strong potent effect on blocking the translocation **(Fig 4)**.

### FQs suppress the expression of TLR4 and phosphorylation of STAT3 in cadmium-activated U-87 MG human astrocytes and PHAs

**Fig 5:**
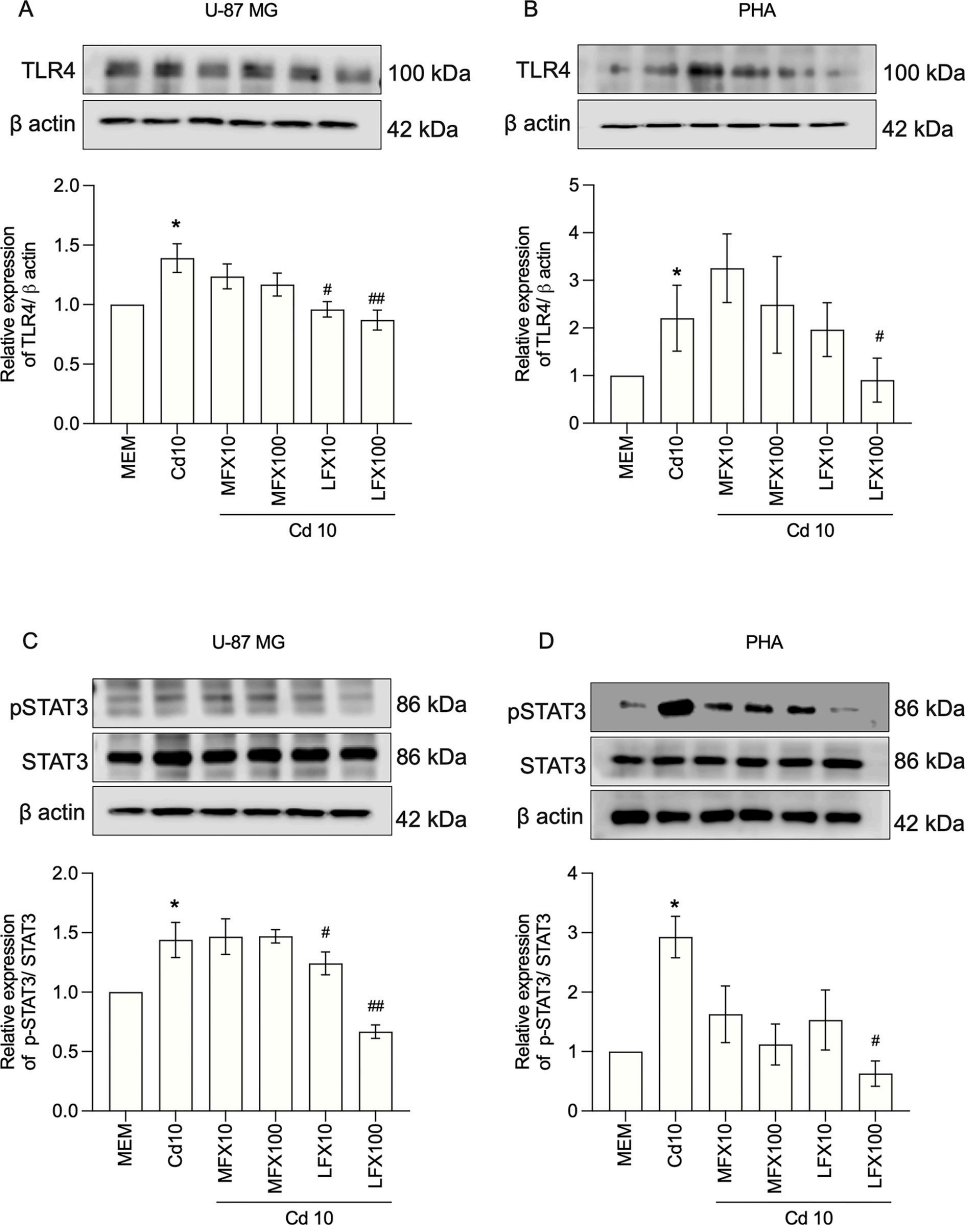
Effect of moxifloxacin and levofloxacin on cadmium-induced TLR4 and STAT3 pathway in U-87 MG human astrocytes and primary human astrocytes. **A, B** Representative blots of TLR4 in U87-MG cells and primary human astrocytes exposed to cadmium (10 μM) in the absence or the presence of 10 and 100 μM moxifloxacin or levofloxacin for 3 h. **C, D** Representative blots of phosphorylated and total forms of STAT3 in U87-MG cells and primary human astrocytes exposed to cadmium (10 μM) in the absence or the presence of 10 and 100 μM moxifloxacin or levofloxacin for 3 h. Data represents mean±SEM of at least three independent experiments. *P<0.05 compared between cadmium-treated cells and mock-treated cells; ^#^P<0.05 and ^##^P<0.01 compared between cells exposed to cadmium and moxifloxacin or cadmium and levofloxacin compared with cadmium alone. Cd, Cadmium; MFX, Moxifloxacin; LFX, Levofloxacin. The original blots are presented in S1 Raw images.

Previous reports demonstrate that the TLR4 pathway mediates the inhibitory effect of FQs on pro-inflammatory factor expression and that FQ is a TLR4 antagonist [27]. Therefore, we determined the effect of TLR4 activation on the inhibitory effects of FQs on inflammatory responses. As shown in **Fig 5A, 5B**, both U-87 MG human astrocytes and PHAs exhibited a marked increase in TLR4 levels in cadmium-treated cells (P < 0.05), a response that was notably diminished in the presence of levofloxacin. This indicates that the ameliorating effects of levofloxacin treatment on cadmium toxicity may stem from its regulation of the TLR4 signaling pathway. Because cross-talk between TLR4 and STAT3 regulates cytokine-dependent inflammation, we next investigated whether STAT3 was also involved in the anti-inflammatory effects of FQs. As shown in **Fig 5C, 5D**, consistent with the results of TLR4, levofloxacin inhibited the STAT3 phosphorylation induced by cadmium. By contrast, moxifloxacin had no inhibitory effect on cadmium-induced TLR4 expression or STAT3 activation in human astrocytes.

### FQ alters transcriptome profiles of cadmium-activated U-87 MG human astrocytes

**Fig 6:**
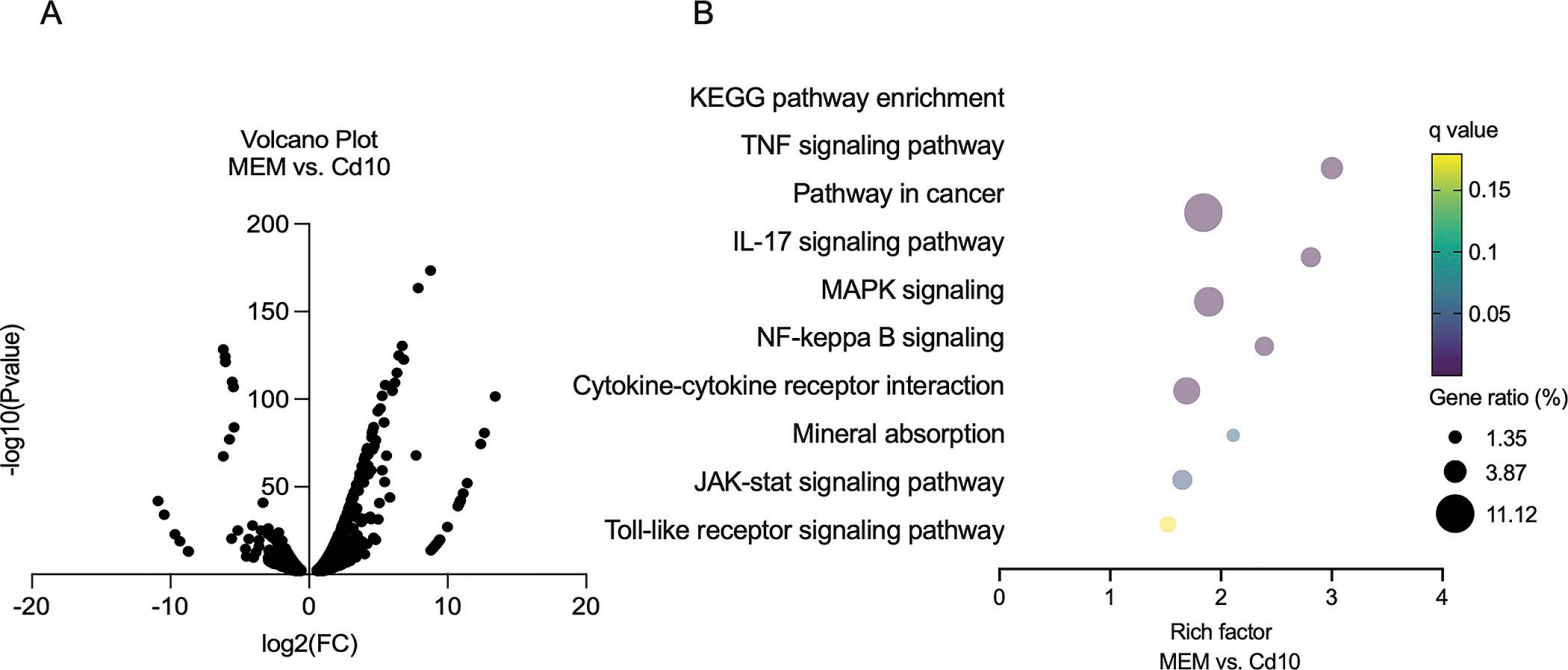
Comparative transcriptomics analysis for differentially expressed genes (DEGs) in cells treated with and without cadmium. **A** Volcano plot of up-regulated (p < 0.05, log2FC > 1) and down-regulated (p < 0.05, log2FC < −1) DEGs. **B** KEGG enrichment scatter plot of up-regulated DEGs. The dot size indicates the amount of DEG enriched in the pathway. The darker the color, the more significant the enrichment result. Cd, Cadmium; FC, Fold change.

To comprehensively understand the effects of FQs on cadmium-induced inflammatory responses in human astrocytes, and to study the underlying mechanisms, we performed whole-transcriptome sequencing (RNA-seq) analysis. Gene expression from cadmium-treated cells was compared with that from mock-treated, cadmium and moxifloxacin-treated, and cadmium and levofloxacin-treated cells. Comparative transcriptome analyses identified 2012 DEGs, including 1268 upregulated and 744 downregulated genes in cadmium-treated cells relative to mock-treated cells **(Fig 6A)**. We next clustered the DEGs and predicted the functional canonical pathway networks associated with these changes in gene expression using Gene Set Enrichment Analysis. Results of KEGG enrichment analysis showed the top-ranked upregulated canonical pathways were “TNF (tumor necrosis factor) signaling pathway,” “pathways in cancer,” “IL-17 signaling pathway,” “MAPK signaling pathway,” “NF-κB signaling pathway,” “cytokine–cytokine receptor interaction,” “mineral absorption,” “JAK (Janus kinase)-STAT signaling pathway,” and “TLR signaling pathway” **(Fig 6B and S1 Table)**. Of note, the enriched KEGG pathways were involved in cadmium-induced neuropathology.

**Fig 7:**
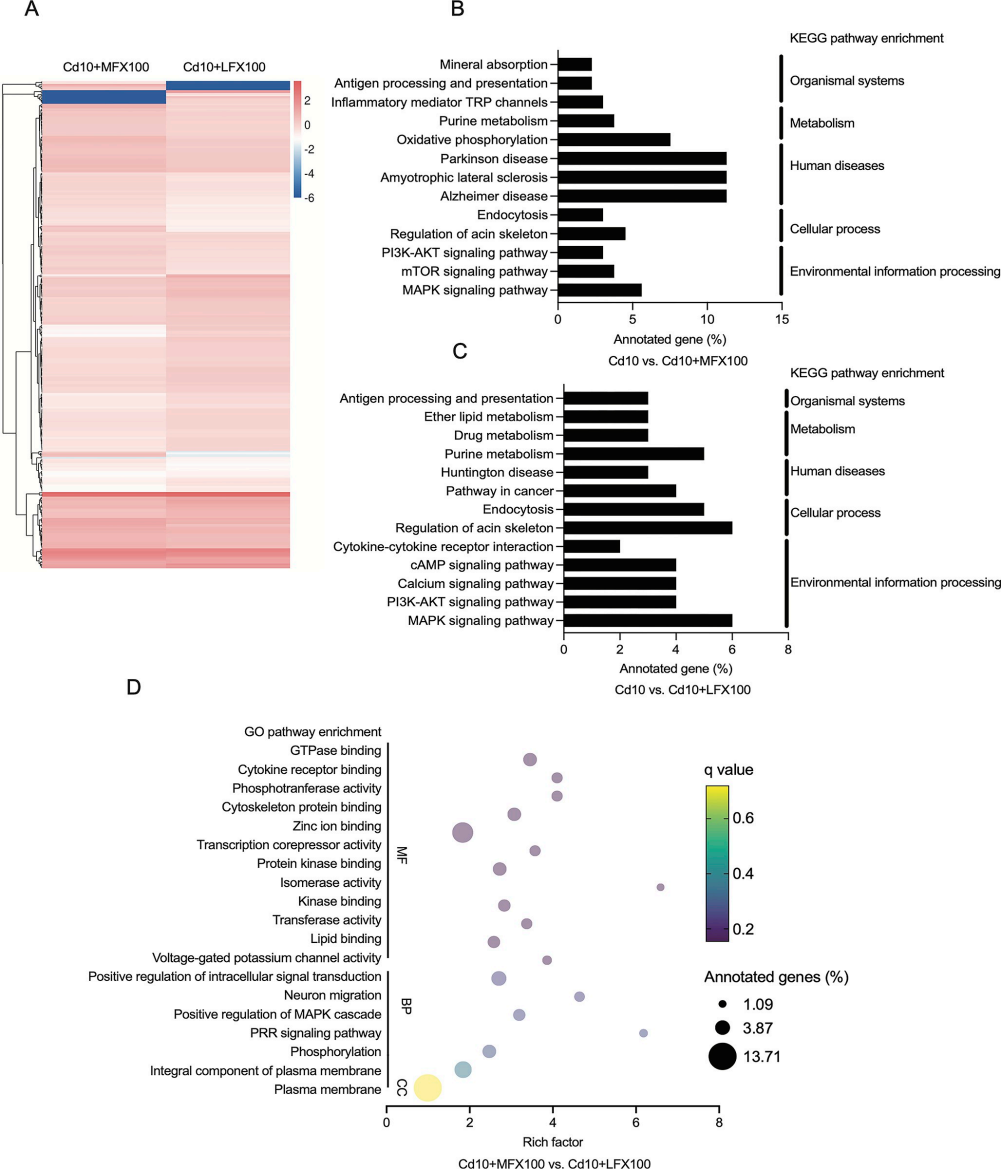
Comparative transcriptomics analysis for differentially expressed genes (DEGs) in cadmium-activated cells after treatment with moxifloxacin or levofloxacin. **A** Heat map and hierarchical clustering of all DEGs in moxifloxacin (Cd10+M100) vs. levofloxacin (Cd10+L100) treatment. Blue indicates downregulated expression, and red indicates upregulated expression compared with a reference expression level. **B, C** KEGG enrichment scatter plot among the most up-regulated and down-regulated DEGs in cadmium-treated cells (Cd10) vs. combinations of moxifloxacin with cadmium-treated cells (Cd10+M100) and cadmium-treated cells (Cd10) vs. combinations of levofloxacin with cadmium-treated cells (Cd10+L100). The bar chart indicates the percentage of annotated genes. **D** Enriched GO terms corresponding to the most up-regulated and down-regulated DEGs in the biological process (BP), cellular component (CC), and molecular function (MF). The dot size indicates the amount of DEG enriched in the pathway. The darker the color, the more significant the enrichment result. Cd, Cadmium; MFX, Moxifloxacin; LFX, Levofloxacin.

When comparing the expression of transcripts in U-87 MG cells modulated by FQs in response to cadmium, a heatmap showed that changes were similar in response to both moxifloxacin and levofloxacin treatment **(Fig 7A)**. Across the two FQs, patterns of DEGs were similarly enriched in the following KEGG pathways: “antigen processing and presentation,” “purine metabolism,” “PI3K (phosphoinositide 3-kinase)-AKT signaling pathway,” “MAPK signaling pathway,” and “inflammatory mediator regulation of TRP (transient receptor potential) channels” **(Fig 7B, 7C and S2 and S3 Tables)**. Nonetheless, some notable differences were observed. KEGG analysis revealed that genes associated with “mineral absorption” and “oxidative stress” pathways were downregulated in moxifloxacin-treated cells, whereas gene signatures in “cytokine–cytokine receptor interaction,” “calcium signaling pathway,” “cAMP signaling pathway,” “NF-κB signaling pathway,” and “JAK-STAT signaling pathway” were downregulated in levofloxacin-treated cells **(S4 Table)**. Results of GO analysis were closely associated with KEGG pathway analysis. As shown in **Fig 7D and S5 Table**, enriched GO terms were mainly related to “cytokine receptor binding,” “zinc ion binding,” “protein kinase binding,” and “phosphotransferase activity,” as well as “MAPK cascade” and “pattern recognition receptor (PRR) signaling pathway.”

## Discussion

The neuroinflammatory effect of cadmium exposure is widely acknowledged. Specifically, our previous study revealed that the pro-inflammatory mediators IL-6 and IL-8 are released from the brain cells after cadmium exposure [8]. Although attenuation of this inflammatory response limits cadmium-induced tissue damage, current therapies for cadmium intoxication are limited and there is no specific treatment targeting cadmium-induced inflammation. Given the health benefits of FQs, we aimed to test the efficacy of repurposing FQs as anti-inflammatory agents. In this study, we compared the anti-inflammatory effects of two classes of FQs in cadmium-treated human astrocytes in vitro. We found that FQ administration, particularly of levofloxacin, ameliorated cadmium-induced inflammatory responses in human astrocytes via reduced activation or expression of inflammation-related signaling molecules, including TLR4, STAT3, ERK, and NF-κB.

The attenuating effects of moxifloxacin and levofloxacin on IL-6 and IL-8 secretion we observed in cadmium-activated astrocytes were achieved at levels close to clinically relevant serum and cerebrospinal fluid concentrations. The human serum and cerebrospinal fluid concentrations of moxifloxacin (following a typical dose of 400 mg) peak at 6.93–13.32 μM and 1.95–2.64 μM, respectively, whereas for levofloxacin (following a typical dose of 750 mg), concentrations are 23.93–36.34 μM and 6.73–11.16 μM, respectively [19]. Comparable to our study, previous reports on the anti-inflammatory characteristics of these two medications were carried out at drug doses ranging from 10 to 200 μM [13, 15]. Interestingly, our results show that moxifloxacin and levofloxacin had a different cytokine inhibition pattern, with levofloxacin having superior anti-inflammatory activity. By contrast, moxifloxacin has more potent immunomodulation activity, which may result from its structure: it has a cyclopropyl moiety at the N1 position of the quinolone core structure, a methoxy group (-CHO) on C8, and a bulky C7 side chain [28]. However, these differences in functionality between levofloxacin and moxifloxacin are not consistent in all studies: in primary human bronchial epithelial cells from patients undergoing lung resections, 20 μM moxifloxacin but not levofloxacin significantly reduced IL-8 release [29]. This suggests that although the anti-inflammatory and immunomodulatory effects of FQs may differ resulting from their chemical structure, which stimulant they treat and the cell model employed may also have an effect.

The inhibition of inflammatory cytokine production by FQs was associated with several molecules involved in inflammatory signaling pathways. The elevation of inflammatory cytokines and phosphorylation of ERK and NF-κB induced by cadmium were downregulated with FQ treatment. We previously found that pretreatment with specific MAPK and NF-κB inhibitors significantly downregulates IL-6 and IL-8 secretion upon cadmium exposure [8]. Consistent with this, downregulation of IL-6 and IL-8 secretion in cadmium-activated astrocytes by moxifloxacin and levofloxacin seemed to be achieved by inhibiting phosphorylation of key intracellular signaling molecules, including ERK and p65 NF-κB. Similarly, moxifloxacin inhibits NF-κB and ERK activation, which is required for IL-8 transcription, in monocytes and bronchial epithelial cells [15, 30, 31], while levofloxacin decreases levels of aerosol MP-376-induced IL-6 and IL-8 by inhibiting NF-κB activation in human cystic fibrosis bronchial epithelial cells [13]. Therefore, the present study extends previous findings that FQs act as inhibitors of NF-κB and ERK signaling pathways.

We found that cadmium upregulated TLR4 expression in human astrocytes and that this was suppressed by FQ administration. TLR4 is a pathogen-associated molecular pattern and damage-associated molecular pattern receptor that plays a crucial role in the innate immune response. Overactivation of TLR4 is closely linked with excessive cytokine production and consequently, heightened immune and inflammatory responses [32, 33]. Cadmium is an alternative TLR4 ligand, which can mediate damage-associated molecular pattern-induced inflammatory responses through PRRs and in turn activate inflammatory signaling [34]. Cadmium exposure causes TLR4 activation-associated cell damage by positively regulating MAPK and NF-κB signaling pathways such as ERK1/2 and p38 MAPK in human airway epithelial cells [35]; MAPK (ERK, JNK, and p38)/NF-κB in porcine and murine testicular cells [36, 37]; and MyD88/NF-κB in chicken peripheral blood lymphocytes [38]. In addition, cadmium activates TLR4 signaling in rat brain cells, leading to the upregulation of neuronal inflammatory cytokines [39]. However, it is yet to be established whether these effects are caused by a physical interaction between TLR4 and cadmium similar to other metal ions: nickel and cobalt trigger TLR4 dimerization and cellular activation through histidine residues in the TLR4 ectodomain [40]. Furthermore, we found that cadmium upregulated TLR4 expression and resulted in activation of its downstream target STAT3 in human astrocytes, which was selectively suppressed by levofloxacin. In support of our findings, a previous study found that treatment with levofloxacin diminished production of IL-1β and tumor necrosis factor-α from lipopolysaccharide-activated rat microglia by blocking lipopolysaccharide binding to TLR4–MD-2; this in turn diminished TLR4 homodimerization and activation [27]. Therefore, TLR4 inhibition may be an alternative pathway by which FQs elicit anti-inflammatory activities.

In our study, pathways enriched in cadmium-treated human astrocytes were closely related to pathways downregulated after FQ treatment. The predominant enriched pathways in cadmium-activated astrocytes were those related to IL-17, MAPK, NF-κB, JAK-STAT, and TLR signaling, as well as cytokine–cytokine receptor interaction and mineral absorption. Our transcriptome analysis data showed that inflammatory pathways were especially enriched in cadmium-treated cells and that treatment with FQs downregulated genes involved in the IL-17 signaling pathway, cytokine–cytokine receptor interaction, and the PRR signaling pathway implicated in their anti-inflammatory role. We also observed a strong chelating effect of moxifloxacin in the presence of cadmium ions. This may be due to its unique structure which contains two main sites (COOH and a C = O group) of divalent metal cation chelate formation [41]. Taken together, results suggest that FQs may serve as an anti-inflammatory agent with chelating actions that could be used in managing cadmium-induced neurotoxicity in the future.

Repurposing FQs for non-infectious diseases such as chronic obstructive pulmonary disease (COPD) (NCT04879030) (ciprofloxacin, levofloxacin, and moxifloxacin) and Crohn’s disease (NCT03850509) (OPS-2071) are still an ongoing clinical trial to carefully evaluated the risk of promoting resistance. Our results demonstrated that moxifloxacin and levofloxacin exhibited neuroprotective effects at concentrations below those typically used for antimicrobial therapy. Minimum inhibitory concentration (MIC) for the majority of pathogens causing bacterial meningitis (Staphylococcus aureus and Streptococcus pneumoniae) and tuberculous meningitis (Mycobacterium tuberculosis) of moxifloxacin and levofloxacin are 0.012 to 2 mg/L (0.03–5 μM) and 0.016 to 8 mg/L (0.04–22 μM), respectively [42]. This implies that neuroprotective concentrations of moxifloxacin and levofloxacin might not have robust antibacterial activity. In addition, a striking concentration-dependent relationship was noticed in the effects of FQs on astrocytes, where elevated concentrations heighten the risk of neurotoxicity. In agreement with several studies, moxifloxacin and levofloxacin can significantly cross BBB [19, 20]. This characteristic enhances their potential to affect the CNS and could lead to both therapeutic benefits and risks of side effects. Therefore, further research is needed to ensure the long-term effectiveness and safety of repurposed antibacterial agents for neuroprotective effects, particularly in vulnerable populations.

Although the present study presents many insights, it also has some limitations. One limitation is that although some experimental studies on the anti-inflammatory effect of FQs were conducted, we did not clarify whether FQs affect other cell signaling pathways, as reported by the RNAseq data, which may also be involved in the anti-inflammatory effect of FQs. It would be interesting for future studies to clarify the multiple targets of moxifloxacin and levofloxacin that mediate its inhibitory effect on pro-inflammatory factor expression. An additional limitation is that we were only able to examine the impact of FQs as a treatment during inflammatory stimulation. Since most cases of cadmium toxicity result in chronically induced inflammation, testing the impact of FQs administered after cadmium-stimulated astrocyte inflammation would be therapeutically important.

## Conclusion

**Fig 8:**
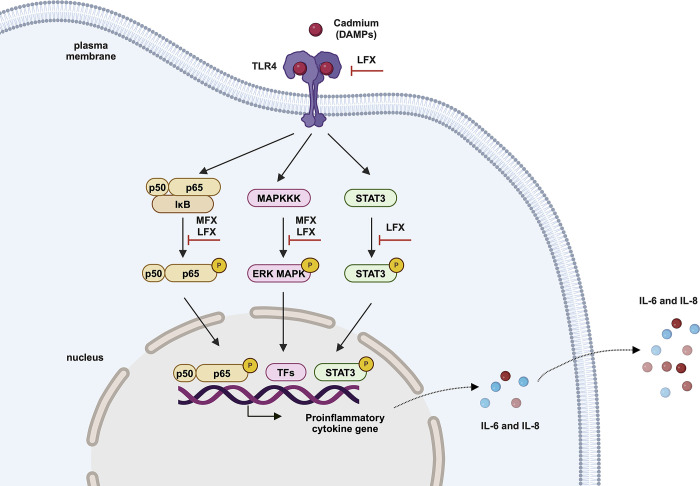
Schematic presentation of possible signaling cascades of fluoroquinolone in suppression of the cadmium-induced inflammatory response. DAMP, damage-associated molecular pattern; MFX, moxifloxacin; LFX, levofloxacin; ERK, extracellular signal-regulated kinases; TLR4, toll-like receptor 4; IL, interleukin; IκB-α, inhibitor of κB-α; p50/p65, nuclear factor-κB; STAT3, signal transducer and activator of transcription 3.

We found that FQs had an inhibitory effect on cadmium-induced release of IL-6 and IL-8 in human astrocytes. Compared to moxifloxacin, levofloxacin appeared to have superior anti-inflammatory properties. Inhibition of TLR4/STAT3 and/or ERK/NF-κB signaling pathways appears to be a key mechanism by which FQs exert their anti-inflammatory effects **(Fig 8)**. FQs may be further applied for anti-inflammatory clinical use; however, further investigations in preclinical and clinical studies are required.

## Supporting information

S1 Table Functional enrichment analysis of DEGs between mock-treated cells (MEM) and cadmium-treated cells (Cd10) by KEGG in human astrocytoma U-87 MG cell lines. (PDF)

S2 Table Functional enrichment analysis of DEGs between cadmium-treated cells (Cd10) and combinations of moxifloxacin with cadmium-treated cells (Cd10+MFX100) by KEGG in human astrocytoma U-87 MG cell lines. (PDF)

S3 Table Functional enrichment analysis of DEGs between cadmium-treated cells (Cd10) and combinations of levofloxacin with cadmium-treated cells (Cd10+LFX100) by KEGG in human astrocytoma U-87 MG cell lines. (PDF)

S4 Table Functional enrichment analysis of DEGs between cadmium-activated cells after treatment with moxifloxacin (Cd10+MFX100) and after treatment with levofloxacin (Cd10+LFX100) by KEGG in human astrocytoma U-87 MG cell lines. (PDF)

S5 Table Functional enrichment analysis of DEGs between cadmium-activated cells after treatment with moxifloxacin (Cd10+MFX100) or levofloxacin (Cd10+LFX100) by GO in human astrocytoma U-87 MG cell lines. (PDF)

S1 Raw images Raw western blot images for Figs 3 and 5. (PDF)
