# Supplementary material for: Anti-inflammatory effects of moxifloxacin and levofloxacin on cadmium-activated human astrocytes: Inhibition of proinflammatory cytokine release, TLR4/STAT3, and ERK/NF-κB signaling pathway
Source: PLoS One. 2025 Jan 14;20(1):e0317281. doi: 10.1371/journal.pone.0317281 (PMC11731778; doi:10.1371/journal.pone.0317281)
Supplement: S1 Table — (PDF) [file pone.0317281.s001.pdf]

Supplementary Table 1

Functional enrichment analysis of DEGs between mock-treated cells (MEM) and cadmium-treated cells (Cd10) by KEGG in human astrocytoma U-87 MG cell lines

Molecular pathway	Pathway ID	KEGG A class	KEGG B class	Focus genes	Intersections
Up-regulated genes					
Pathway in cancer	hsa05200	Human Diseases	Cancer: overview	69	BIRC3;KITLG;PTGER3;GSTO2;PTGS2;GLI2;TRAF4;EDN1;HSP90AA1;MECOM;TGFB2;HSP90AB1;ABL1;GADD45B;HMOX1;HIF1A;BDKRB1;JAG1;XIAP;IL7;NFKB1;CCND1;BIRC2;E2F3;FGF1;HES1;GADD45A;SLC2A1;PIK3R3;PGF;NCOA3;BMP2;WNT2B;COL4A2;ETS1;HEY2;EGLN1;EDNRB;IL6;MYC;FGF5;FGF2;FGF7;IGF1R;PMAIP1;NOTCH1;VEGFC;FOXO1;RUNX1;TGFA;IL15;DAPK3;IL7R;IL12A;ZBTB49;CXCL8;FOS;PTGER4;BCL2;STAT5B;TRAF6;ZNF408;FZD8;JUN;MMP1;PDGFA;TXNRD1;RASSF5;NKX3-1
TNF signaling pathway	hsa04668	Environmental Information Processing	Signal transduction	27	CFLAR;BIRC3;PTGS2;EDN1;CXCL2;JAG1;MAP3K8;CCL2;NFKB1;BIRC2;PIK3R3;TNFAIP3;IL1B;ATF4;LIF;IL6;CREB5;MMP3;VEGFC;CXCL3;CXCL5;IL15;FOS;JUNB;CEBPB;JUN;SOCS3
IL-17 signaling pathway	hsa04657	Organismal Systems	Immune system	24	PTGS2;TRAF4;HSP90AA1;CXCL2;HSP90AB1;CSF3;CCL2;NFKB1;TNFAIP3;IL1B;FOSB;JUND;IL6;MMP3;CXCL3;CXCL5;CXCL8;FOS;CEBPB;IL17D;TRAF6;FOSL1;JUN;MMP1
MAPK signaling pathway	hsa04010	Environmental Information Processing	Signal transduction	48	KITLG;MEF2C;MECOM;TGFB2;GADD45B;HSPB1;MAP3K8;NFKB1;HSPA8;DUSP16;SASH1;SRF;FGF1;IL1R1;GADD45A;PGF;DUSP1;DUSP4;NR4A1;EREG;IL1B;ATF4;JUND;NGF;MYC;DUSP5;FGF5;FGF2;DUSP6;FGF7;IGF1R;EPHA2;DUSP10;VEGFC;RASA2;MRAS;TGFA;FOS;HSPA6;TRAF6;DDIT3;BDNF;JUN;DUSP8;IL1RAP;PDGFA;HSPA1B;HSPA1A
NF-kB signaling pathway	hsa04064	nmental Information Proc	Signal transduction	20	CFLAR;BIRC3;PTGS2;CXCL2;GADD45B;XIAP;NFKB1;BIRC2;IL1R1;GADD45A;TNFAIP3;IL1B;TICAM1;TLR4;TNFRSF11A;BCL10;CXCL3;CXCL8;BCL2;TRAF6
Cytokine-cytokine receptor interaction	hsa04060	Environmental Information Processing	Signaling molecules and interaction	37	CCL26;RELT;CXCL2;TGFB2;IL11;IL21R;IL7;CSF3;CCL2;IL1R1;IL1RL1;INHBA;IL1B;TNFSF9;BMP2;LIF;GDF15;NGF;IL6;IL1RN;IL33;TNFRSF11A;TSLP;BMP6;TNFSF13;IL24;CXCL3;CXCL5;IL15;TNFRSF11B;IL7R;IL12A;CXCL8;IL17D;CLCF1;IL1RAP;CCL3
Mineral absorption	hsa04978	Organismal Systems	Digestive system	12	ATP2B1;HMOX1;VDR;MT2A;ATP1B1;MT1B;MT1E;SLC30A1;SLC26A9;MT1F;MT1A;MT1M
Toll-like receptor signaling pathway	hsa04620	Organismal Systems	Immune system	13	MAP3K8;NFKB1;PIK3R3;IL1B;TICAM1;IL6;TLR4;IL12A;CXCL8;FOS;TRAF6;JUN;CCL3
JAK-STAT signaling pathway	hsa04630	Environmental Information Processing	Signal transduction	23	IL11;IL21R;IL7;CSF3;CCND1;PIK3R3;SOCS2;LIF;IL6;MYC;MCL1;TSLP;IL24;IL15;IL7R;IL12A;SOCS6;BCL2;IL17D;STAT5B;SOCS3;PDGFA;IRF9
Down-regulated genes					
Cell cycle	hsa04110	Cellular Processes	Cell growth and death	22	DBF4;E2F2;MCM2;ORC1;CDC6;CDC25B;CCNE1;CDK6;CDKN1B;TTK;CDC20;TGFB3;PCNA;CCNB1;CCNA2;SKP2;BUB1B;CCNB2;CDC25C;CDC25A;PLK1;CCNE2
p53 signaling pathway	hsa04115	Cellular Processes	Cell growth and death	10	GTSE1;SESN1;WDR76;CCNE1;BBC3;CDK6;CCNB1;CCNB2;PPM1D;CCNE2
