# Supplementary material for: Anti-inflammatory effects of moxifloxacin and levofloxacin on cadmium-activated human astrocytes: Inhibition of proinflammatory cytokine release, TLR4/STAT3, and ERK/NF-κB signaling pathway
Source: PLoS One. 2025 Jan 14;20(1):e0317281. doi: 10.1371/journal.pone.0317281 (PMC11731778; doi:10.1371/journal.pone.0317281)
Supplement: S2 Table — (PDF) [file pone.0317281.s002.pdf]

Supplementary Table 2

Functional enrichment analysis of DEGs between cadmium-treated cells (Cd10) and combinations of moxifloxacin with cadmium-treated cells (Cd10+MFX100) by KEGG in human astrocytoma U-87 MG cell lines

Molecular pathway	Pathway ID	KEGG A class	KEGG B class	Focus genes	Intersections
Up-regulated genes					
MAPK signaling pathway	hsa04010	Environmental Information Processing	Signal transduction	6	CDC42;MAP3K1;DDIT3;CACNB4;PLA2G4B;AL031281.2
Mineral absorption	hsa04978	Organismal Systems	Digestive system	3	MT1B;MT1A;MT1M
Down-regulated genes					
Oxidative phosphorylati on	hsa00190	Metabolism	Energy metabolism	10	MT-ND6;MT-CYB;MT-ND5;MT-CO1;MT-ND4;MT-ATP6;MT-CO3;MT-ND4L;MT-ATP8;AC005943.1
Up- and down-regulated genes					
Alzheimer disease	hsa05010	Human Diseases	Neurodegenerative disease	14	TUBB2B;AXIN2;DDIT3;MT-ND6;MT-CYB;MT-ND5;MT-CO1;MT-ND4;MT-ATP6;MT-CO3;MT-ND4L;MT-ATP8;AL139300.1;AC005943.1
Amyotrophic lateral sclerosis	hsa05014	Human Diseases	Neurodegenerative disease	14	HSPA5;TUBB2B;NRG2;DDIT3;MT-ND6;MT-CYB;MT-ND5;MT-CO1;MT-ND4;MT-ATP6;MT-CO3;MT-ND4L;MT-ATP8;AL139300.1;AC005943.1
Parkinson disease	hsa05012	Human Diseases	Neurodegenerative disease	14	HSPA5;TUBB2B;DDIT3;MT-ND6;MT-CYB;MT-ND5;MT-CO1;MT-ND4;MT-ATP6;MT-CO3;MT-ND4L;MT-ATP8;AL139300.1;AC005943.1
Regulation of acin skeleton	hsa04810	Cellular Processes	Cell motility	5	CDC42;BDKRB1;CCDC125;AC004922.1;AL031281.2
Purine metabolism	hsa00230	Metabolism	Nucleotide metabolism	5	NME1-NME2;PDE4C;PDE1C;PDE3A;AL022238.4
mTOR signaling pathway	hsa04150	nmental Information Proc	Signal transduction	4	SESN2;RASGEF1B;DDIT4;CASTOR1
PI3K-AKT signaling pathway	hsa04151	nmental Information Proc	Signal transduction	3	BCL2L11;DDIT4;MTCP1
Endocytosis	hsa04144	Cellular Processes	Transport and catabolism	3	CDC42;MVB12B;AC004922.1;AL031281.2
Inflammatory mediator regulation TRP channels	hsa04750	Organismal Systems	Sensory system	3	BDKRB1;CYP2J2;TRPV1;PLA2G4B
Antigen processing and presentation	hsa04612	Organismal Systems	Immune system	2	HSPA5;AL669918.1
