# Supplementary material for: Anti-inflammatory effects of moxifloxacin and levofloxacin on cadmium-activated human astrocytes: Inhibition of proinflammatory cytokine release, TLR4/STAT3, and ERK/NF-κB signaling pathway
Source: PLoS One. 2025 Jan 14;20(1):e0317281. doi: 10.1371/journal.pone.0317281 (PMC11731778; doi:10.1371/journal.pone.0317281)
Supplement: S3 Table — (PDF) [file pone.0317281.s003.pdf]

Supplementary Table 3

Functional enrichment analysis of DEGs between cadmium-treated cells (Cd10) and combinations of levofloxacin with cadmium-treated cells (Cd10+LFX100) by KEGG in human astrocytoma U-87 MG cell lines

Molecular pathway	Pathway ID	KEGG A class	KEGG B class	Focus genes	Intersections
Down-regulated genes					
Ether lipid metabolism	hsa00565	Metabolism	Lipid metabolism	3	TMEM189-UBE2V1;HRASLS;PLA2G4B
Cytokine-cytokine	hsa04060	Environmental Information Processing	Signaling molecules and interaction	2	CXCL12;TNFRSF6B
Antigen processing and presentation	hsa04612	Organismal Systems	Immune system	1	HSPA1L
Up- and down-regulated genes					
Regulation of acin skeleton	hsa04810	Cellular Processes	Cell motility	5	CDC42;CXCL12;ITGA4;AC004922.1;AL031281.2
Endocytosis	hsa04144	Cellular Processes	Transport and catabolism	5	CDC42;PARD6B;HSPA1L;AC004922.1;AL031281.2
Purine metabolism	hsa00230	Metabolism	Nucleotide metabolism	5	NME1-NME2;PDE4C;LRGUK;AL022238.4;AC008397.2
MAPK signaling	hsa04010	Environmental Information Processing	Signal transduction	4	CDC42;HSPA1L;PLA2G4B;AL031281.2
Calcium signaling	hsa04020	Environmental Information Processing	Signal transduction	4	GNAL;OXTR;P2RX5-TAX1BP3
cAMP signaling pathway	hsa04024	Environmental Information Processing	Signal transduction	4	PDE4C;CNGA3;OXTR;AC008397.2
PI3K-AKT signaling pathway	hsa04151	Environmental Information Processing	Signal transduction	3	ITGA4;DDIT4;MTCP1
Pathway in cancer	hsa05200	Human Diseases	Cancer: overview	3	GSTO2;BBC3;CXCL12
Huntington disease	hsa05016	Human Diseases	Neurodegenerative disease	3	BBC3;AC018523.2;AC105052.3
Drug metabolism	hsa00983	Metabolism	Xenobiotics biodegradation and metabolism	2	NME1-NME2;GSTO2
