# Supplementary material for: Anti-inflammatory effects of moxifloxacin and levofloxacin on cadmium-activated human astrocytes: Inhibition of proinflammatory cytokine release, TLR4/STAT3, and ERK/NF-κB signaling pathway
Source: PLoS One. 2025 Jan 14;20(1):e0317281. doi: 10.1371/journal.pone.0317281 (PMC11731778; doi:10.1371/journal.pone.0317281)
Supplement: S4 Table — (PDF) [file pone.0317281.s004.pdf]

Supplementary Table 4

Functional enrichment analysis of DEGs between cadmium-activated cells after treatment with moxifloxacin (Cd10+MFX100) and after treatment with levofloxacin (Cd10+LFX100) by KEGG in human astrocytoma U-87 MG cell lines

Molecular pathway	Pathway ID	KEGG A class	KEGG B class	Focus genes	Intersections
Up-regulated genes					
Amyotrophic lateral sclerosis	hsa05014	Human Diseases	Neurodegenerative disease	9	SLC1A2;TUBB2B;DDIT3;MT-ND6;MT-ND5;MT-ND4L;AC011448.1;AL163636.2;AC005943.1
Alzheimer disease	hsa05010	Human Diseases	Neurodegenerative disease	8	TUBB2B;DDIT3;AD000671.1;MT-ND6;MT-ND5;MT-ND4L;AC011448.1;AC005943.1
Parkinson disease	hsa05012	Human Diseases	Neurodegenerative disease	8	TUBB2B;DDIT3;SEPT5;MT-ND6;MT-ND5;MT-ND4L;AC011448.1;AC005943.1
Oxidative phosphorylati	hsa00190	Metabolism	Energy metabolism	5	MT-ND6;MT-ND5;MT-ND4L;AC011448.1;AC005943.1
MAPK signaling	hsa04010	Environmental Information Processing	Signal transduction	2	FGF18;DDIT3
Down-regulated genes					
JAK-STAT signaling	hsa04630	Environmental Information Processing	Signal transduction	3	SOCS2;GFAP;IRF9
cAMP signaling	hsa04024	Environmental Information Processing	Signal transduction	3	ADCYAP1R1;NFKBIA;CNGA3
Calcium signaling pathway	hsa04020	Environmental Information Processing	Signal transduction	3	HTR2A;GNAL;PDE1C
NF-kB signaling pathway	hsa04064	Environmental Information Processing	Signal transduction	2	NFKBIA;CXCL12
Cytokine-cytokine [illegible]	hsa04060	Environmental Information Processing	Signaling molecules and interaction	1	CCL2
Mineral absorption	hsa04978	Organismal Systems	Digestive system	1	MT1A
