# Supplementary material for: Anti-inflammatory effects of moxifloxacin and levofloxacin on cadmium-activated human astrocytes: Inhibition of proinflammatory cytokine release, TLR4/STAT3, and ERK/NF-κB signaling pathway
Source: PLoS One. 2025 Jan 14;20(1):e0317281. doi: 10.1371/journal.pone.0317281 (PMC11731778; doi:10.1371/journal.pone.0317281)
Supplement: S5 Table — (PDF) [file pone.0317281.s005.pdf]

Supplementary Table 5

Functional enrichment analysis of DEGs between cadmium-activated cells after treatment with moxifloxacin (Cd10+MFX100) or levofloxacin (Cd10+LFX100) by GO in human astrocytoma U-87 MG cell lines

Molecular pathway	GO ID	Focus genes	Intersections
Molecular function			
GTPase binding	GO:0051020	6	CDKL5;PIH1D2;RANBP3L;PIFO;AC008878.3;TBC1D3L
Cytokine receptor binding	GO:0005126	4	DDTL;DDT;CXCL12;TICAM2
Phosphotransferase activity, alcohol group as acceptor	GO:0016773	4	CDKL5;MAP3K1;PIP5KL1;RBKS
Cytoskeletal protein binding	GO:0008092	6	MAP3K1;BCL2L11;TOR2A;PIFO
Zinc ion binding	GO:0008270	14	ESR1;MMP15;DHX58;TRIM2;IFIH1;PAPPA2;ADAMTS14;NEIL1;PTER;ZFAND2A;TCEA3;TRIM59;AC079594.2;AC005324.3
Protein kinase binding	GO:0019901	6	MAP3K1;TRIB3;BCL2L11;DUSP19;PIFO;DOK7
Isomerase activity	GO:0016853	2	DDTL;DDT
Kinase binding	GO:0019900	5	MAP3K1;GFAP;BCL2L11;DUSP19;PIFO
Transferase activity, transferring phosphorus-containing groups	GO:0016772	4	CDKL5;MAP3K1;PIP5KL1;RBKS
Lipid binding	GO:0008289	5	MAP3K1;SYTL5;APOLD1;PSG4;TICAM2
Voltage-gated potassium channel activity	GO:0005249	3	KCNC3;KCNQ3;KCNQ5
Biological process			
Positive regulation of intracellular signal transduction	GO:1902533	6	MAP3K1;DDTL;DDT;BCL2L11;DUSP19;DDIT3
Neuron migration	GO:0001764	3	CDKL5;CXCL12;TUBB2B
Positive regulation of MAPK cascade	GO:0043410	4	MAP3K1;DDTL;DDT;DUSP19
Pattern recognition receptor signaling pathway	GO:0002221	2	MAP3K1;TICAM2
Phosphorylation	GO:0016310	5	CDKL5;MAP3K1;DUSP19;PIP5KL1;RBKS
Cellular component			
Integral component of plasma membrane	GO:0005887	9	HTR2A;MMP15;SLC16A7;GPR75;AMIGO1;PSG4;TICAM2;P2RX5-TAX1BP3
Plasma membrane	GO:0005886	24	NFKBIA;CXCL12;GPR75;SLC14A1;RGS16;SYTL5;FMNL3;HARBI1;AMIGO1;HYLS1;TMEFF1;PSG4;TICAM2;MSANTD3-TMEFF1;AC005324.3;PCDHGA8;PCDHGA10;PCDHGB2;PCDHGB7;LIMS4;RPL36A-HNRNPH2;PCDHGA9;AC008878.3
