# Supplementary material for: Anti-inflammatory effects of moxifloxacin and levofloxacin on cadmium-activated human astrocytes: Inhibition of proinflammatory cytokine release, TLR4/STAT3, and ERK/NF-κB signaling pathway
Source: PLoS One. 2025 Jan 14;20(1):e0317281. doi: 10.1371/journal.pone.0317281 (PMC11731778; doi:10.1371/journal.pone.0317281)

Images of Western Blotting for Figure 3A and 3C

U-87 MG (N1)

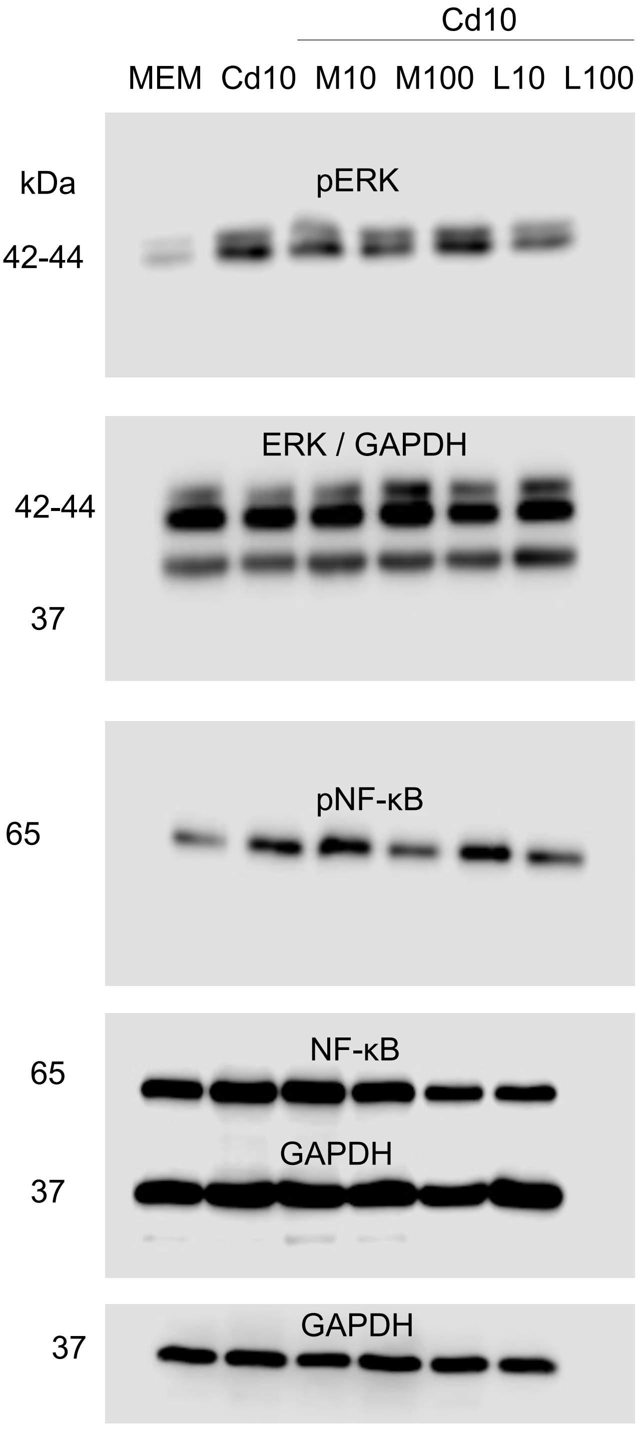

U-87 MG (N2)

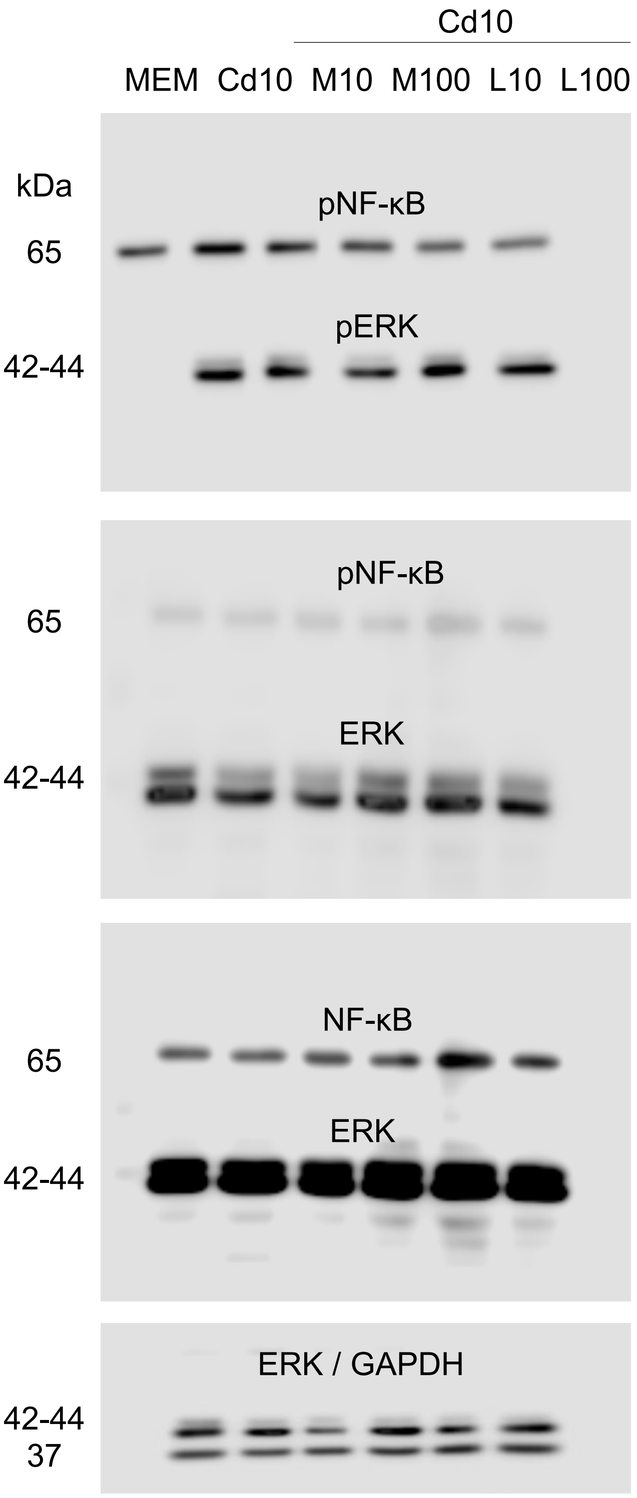

Images of Western Blotting for Figure 3A and 3C

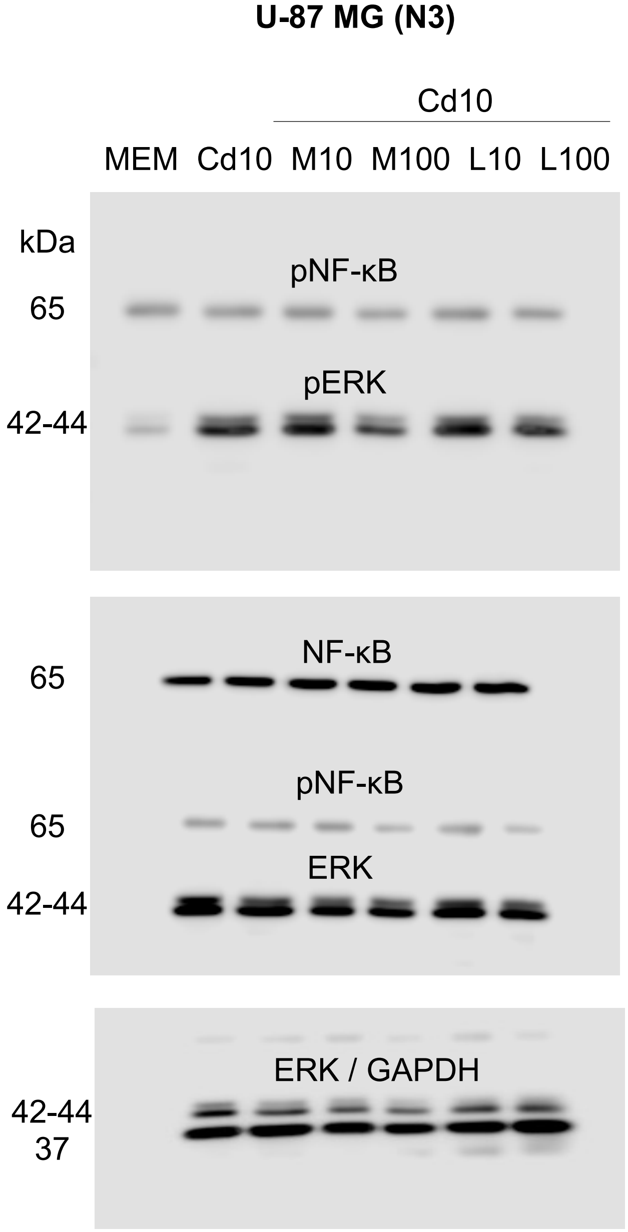

Images of Western Blotting of Figure 3B and 3D

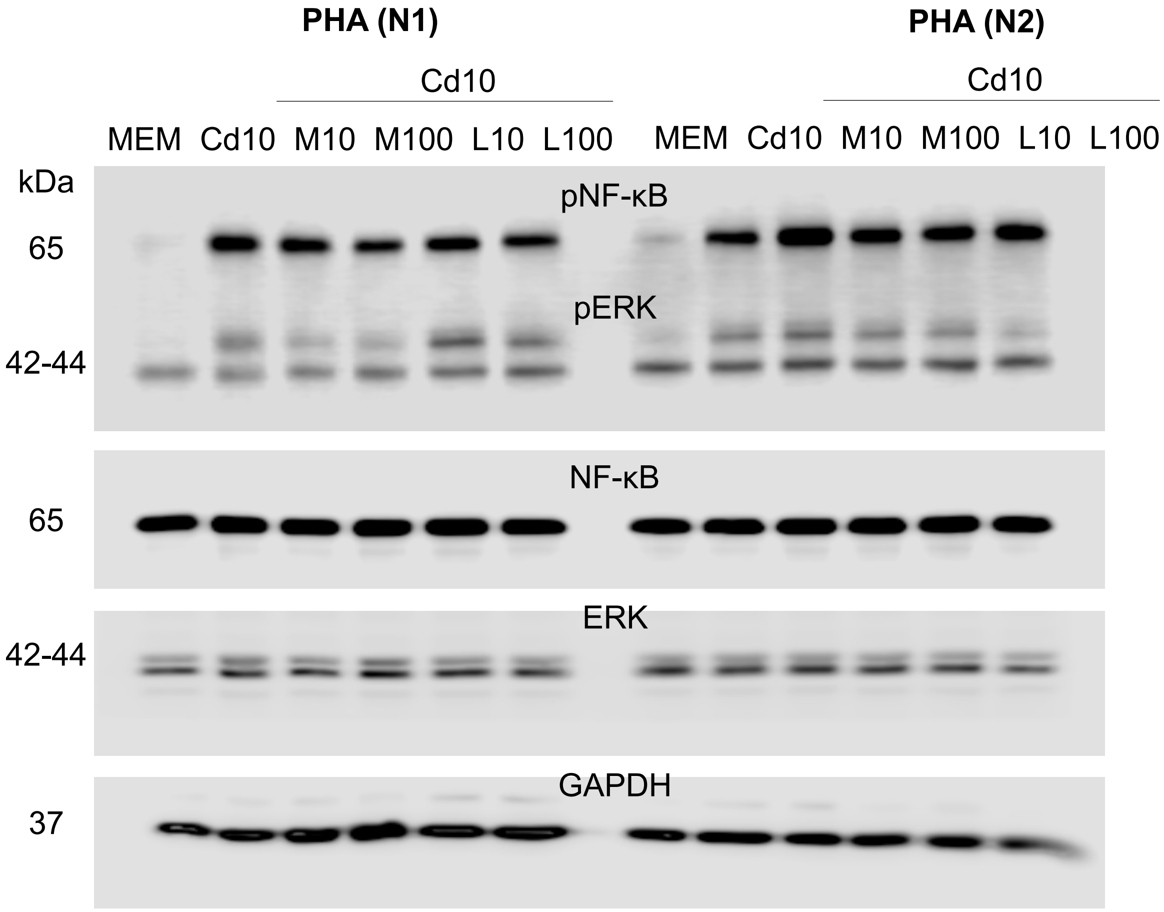

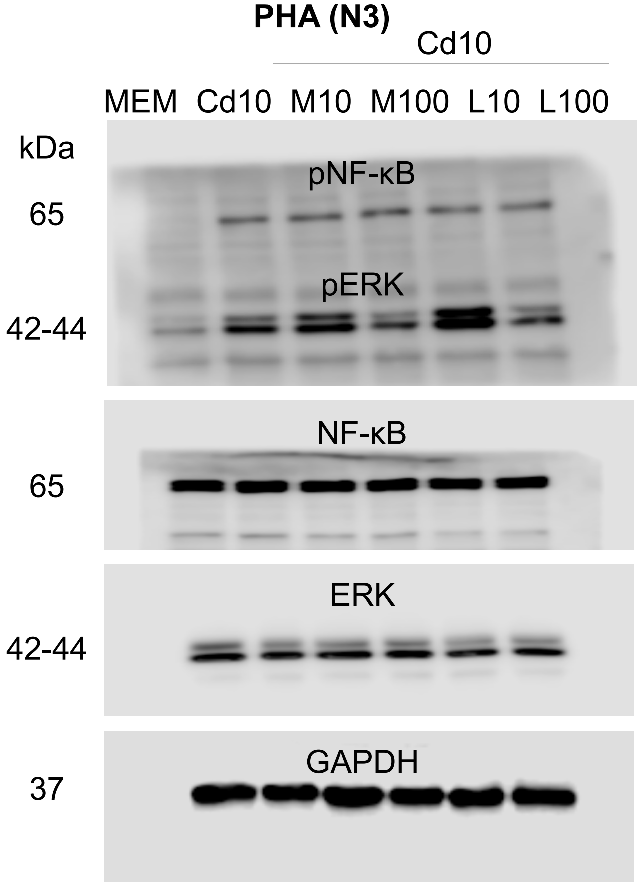

Images of Western Blotting for Figure 5A and 5C

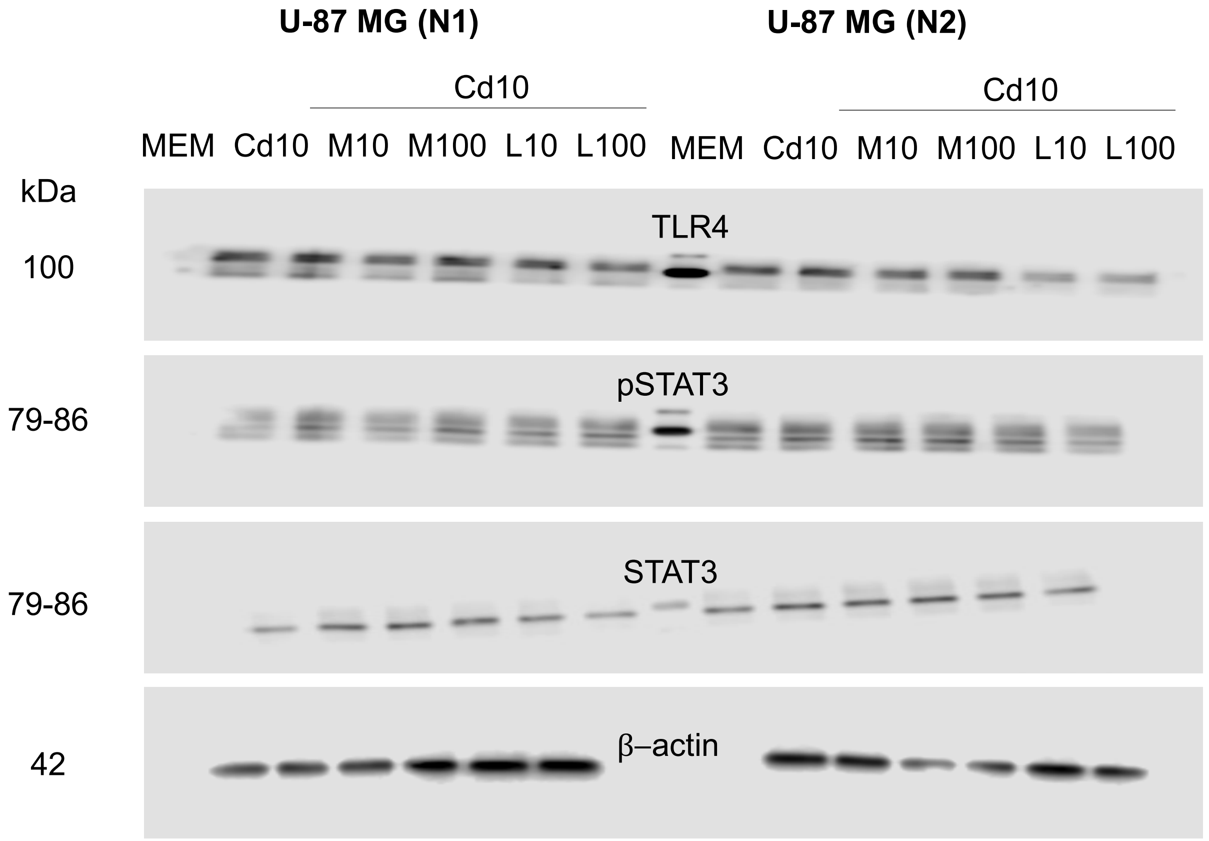

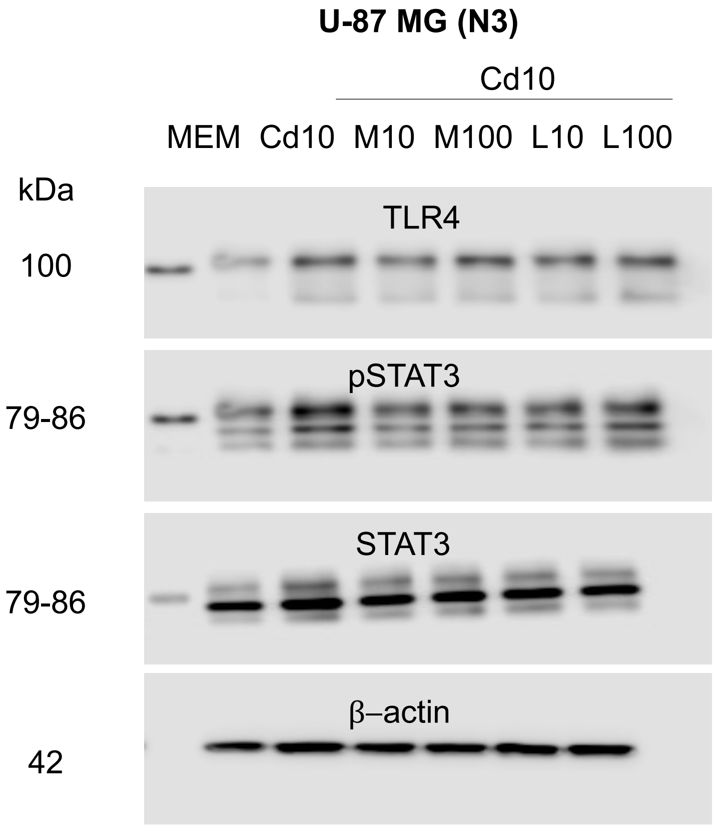

Images of Western Blotting for Figure 5B and 5D

PHA (N1)

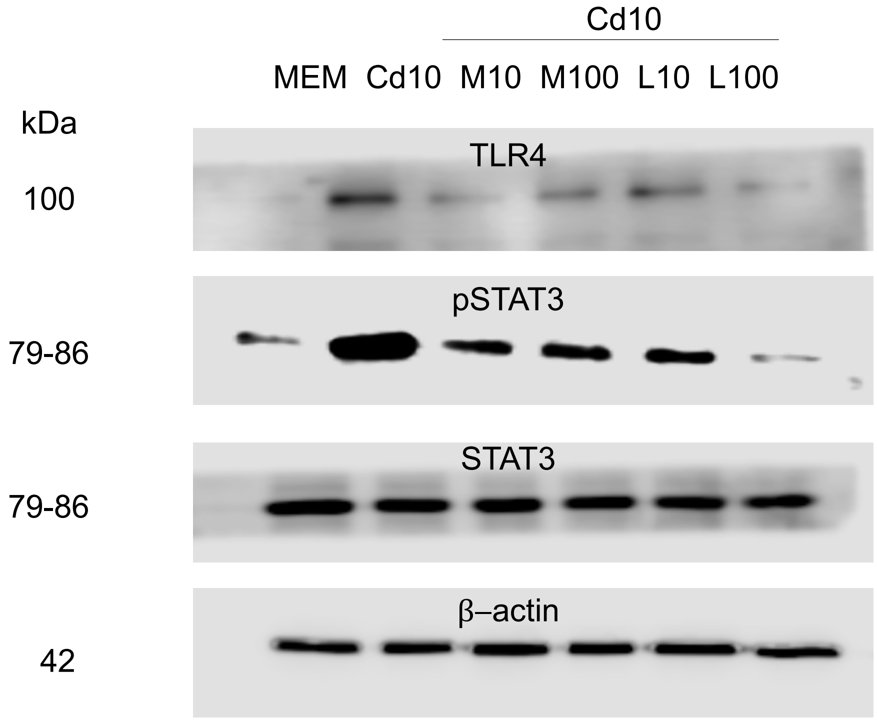

PHA (N2)

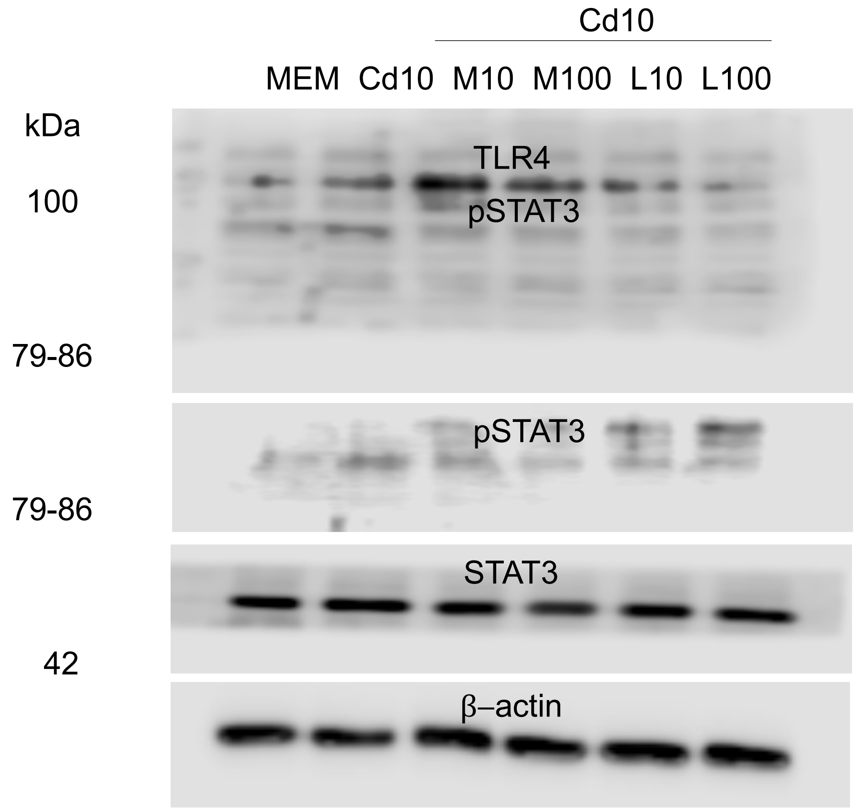

Images of Western Blotting for Figure 5B and 5D

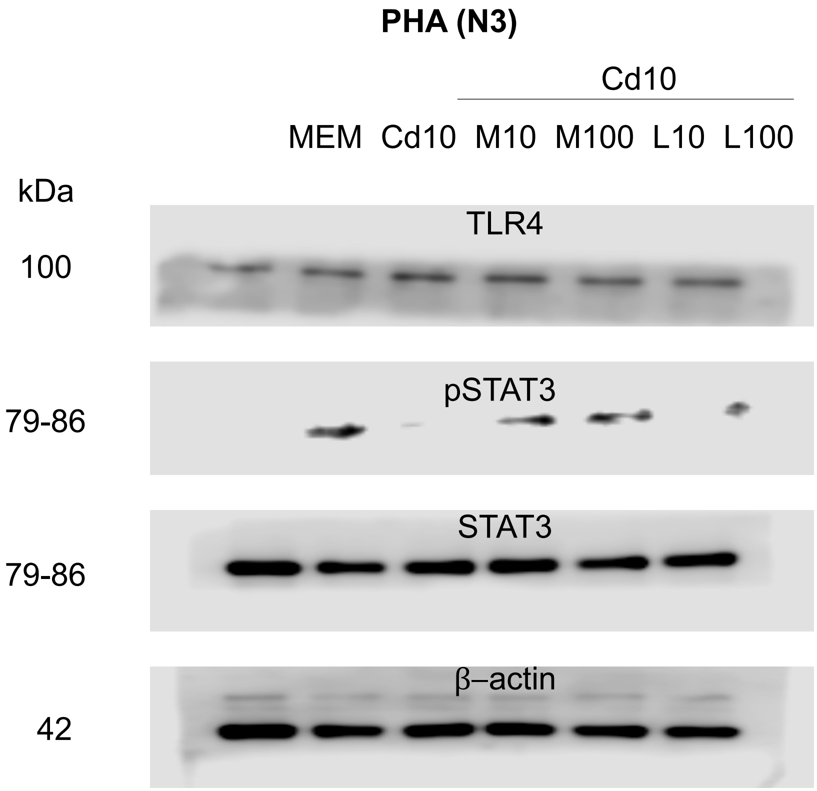

Supplement: S1 Raw images — (PDF) [file pone.0317281.s006.pdf]
